# Antibiotic dosing recommendations in critically ill patients receiving new innovative kidney replacement therapy

**DOI:** 10.1186/s12882-024-03469-2

**Published:** 2024-02-27

**Authors:** Susan J. Lewis, Bruce A. Mueller

**Affiliations:** 1 Department of Pharmacy Practice, College of Pharmacy, University of Findlay, 1000 N. Main Street, 45840 Findlay, OH USA; 2 Department of Pharmacy, Mercy Health - St. Anne Hospital, 43623 Toledo, OH USA; 3 Clinical Pharmacy Department, College of Pharmacy, University of Michigan, MI 48109 Ann Arbor, USA

**Keywords:** Antibiotics, Beta-lactams, Monte Carlo simulation, Critically-ill, Pharmacokinetics/pharmacodynamics, Tablo kidney replacement therapy

## Abstract

**Background:**

The Tablo Hemodialysis System is a new innovative kidney replacement therapy (KRT) providing a range of options for critically ill patients with acute kidney injury. The use of various effluent rate and treatment durations/frequencies may clear antibiotics differently than traditional KRT. This Monte Carlo Simulation (MCS) study was to develop antibiotic doses likely to attain therapeutic targets for various KRT combinations.

**Methods:**

Published body weights and pharmacokinetic parameter estimates were used to predict drug exposure for cefepime, ceftazidime, imipenem, meropenem and piperacillin/tazobactam in virtual critically ill patients receiving five KRT regimens. Standard free β-lactam plasma concentration time above minimum inhibitory concentration targets (40–60%*f*T_> MIC_ and 40–60%*f*T_> MICx4_) were used as efficacy targets. MCS assessed the probability of target attainment (PTA) and likelihood of toxicity for various antibiotic dosing strategies. The smallest doses attaining PTA ≥ 90% during 1-week of therapy were considered optimal.

**Results:**

MCS determined β-lactam doses achieving ∼90% PTA in all KRT options. KRT characteristics influenced antibiotic dosing. Cefepime and piperacillin/tazobactam regimens designed for rigorous efficacy targets were likely to exceed toxicity thresholds.

**Conclusion:**

The flexibility offered by new KRT systems can influence β-lactam antibiotic dosing, but doses can be devised to meet therapeutic targets. Further clinical validations are warranted.

**Supplementary Information:**

The online version contains supplementary material available at 10.1186/s12882-024-03469-2.

## Background

Sepsis remains the primary cause of death in critically ill patients requiring kidney replacement therapy (KRT) [1, 2]. KRT affects the pharmacokinetics (PK) and dosing of most antibiotic agents. While many reviews have been published outlining antibiotic dosing adjustments for standard KRTs [3–5], new KRT options are being used in practice without supporting dosing guidance. These new KRT regimens utilize different treatment frequencies, durations, and blood/dialysate/ultrafiltrate flow rates, and offer clinicians treatment flexibility to meet critically ill patients’ individual needs [6, 7]. However, some of these new KRT frequency/duration/flow rate combinations are likely to remove antibiotics differently than standard thrice-weekly intermittent hemodialysis or continuous KRT, requiring antibiotic dosage adjustment to attain meet therapeutic targets. It is not feasible to conduct clinical pharmacokinetic trials for all KRT options for all commonly used antibiotics in these patients. Consequently, Monte Carlo Simulations (MCS) that replicate various KRT regimens and that use published demographic and PK data derived from critically ill patients receiving KRT can be conducted to determine which dosing regimens are likely to meet therapeutic targets while minimizing the risk of toxicity [8–12]. This study was to predict optimal doses of five β-lactam antibiotics for various KRT regimens utilized in new innovative KRT systems.

## Methods

### Development of mathematical pharmacokinetic model

**Fig. 1:**
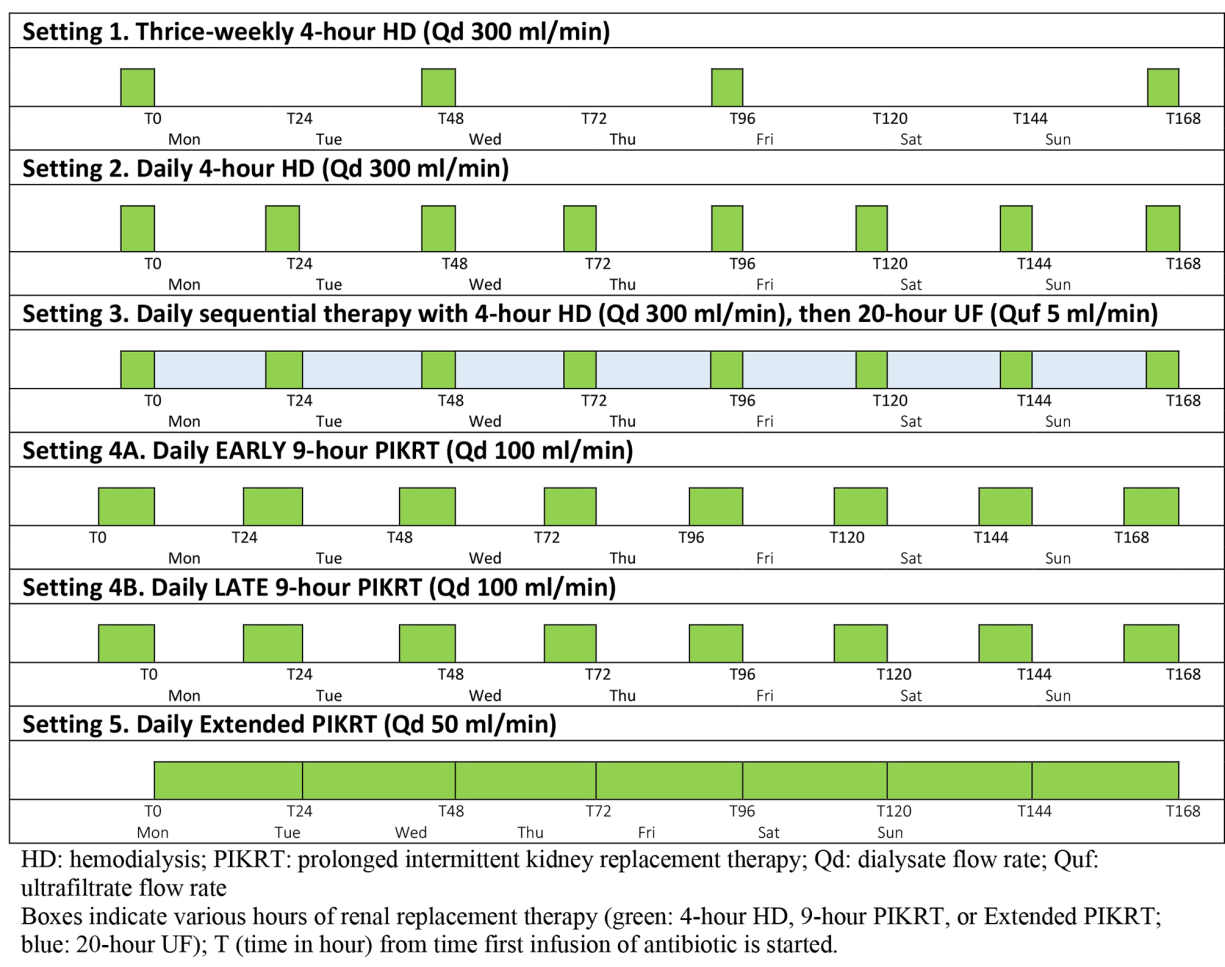
Five simulated kidney replacement therapy settings

One compartment, first order PK models were developed to predict drug exposure of five β-lactam antibiotics (i.e. cefepime, ceftazidime, imipenem, meropenem and piperacillin/tazobactam) in virtual critically ill patients receiving KRT. Input parameters integrated into PK models were outlined in Table 1. Patient body weights were obtained from a large trial involving critically ill patients undergoing KRT [13] and the PK parameters with variances (i.e. standard deviation) were derived from pertinent PK studies on each study drug conducted in critically ill patients receiving KRT [14–41]. Figure 1 depicts five simulated KRT settings: (1) thrice-weekly (Mon-Wed-Fri) 4-hour hemodialysis (HD) at dialysate flow rate (Qd) 300 ml/min, (2) daily 4-hour HD at Qd 300 ml/min, (3) daily sequential therapy consisting of 4-hour HD at Qd 300 ml/min followed by 20-hour ultrafiltration (UF) at ultrafiltrate flow rate (Quf) 5 ml/min, (4) daily 9-hour prolonged intermittent kidney replacement therapy (PIKRT) at Qd 100 ml/min, and (5) daily 24-hour extended PIKRT at Qd 50 ml/min. Transmembrane drug clearance in HD and UF is a function of effluent flow rate (i.e. Qd or Quf) and extraction coefficient. Regression analyses were performed utilizing published transmembrane drug clearance at various effluent flow rates [14–28, 31–36, 38, 41–63]. The best fitting relationships were modeled to extrapolate extraction coefficient (i.e. saturation or sieving coefficient) at the desired effluent flow rates in KRT settings. Patients were assumed to be anuric adults with no residual renal function. Log-Gaussian distribution was assumed for all input parameters. The equations used in the PK model were as follows:

**Table 1:** Demographic and pharmacokinetic parameters used in PK models

		Cefepime	Ceftazidime	Imipenem	Meropenem	Piperacillin	Tazobactam
Body weight (kg)		88 ± 26 [40–177]^13^					
Volume of distribution (L/kg)		0.45 ± 0.25 [0.25–1.11]^14–17^	0.29 ± 0.20 [0.17–1.10]^18–21^	0.36 ± 0.15 [0.11–0.75]^22–28^	0.39 ± 0.18 [0.08–1.07]^31–36^	0.4 ± 0.21 [0.12–1.72]^38–40^	0.5 ± 0.37 [0.11–2.13]^40^
Unbound fraction of drug		0.79 ± 0.09 [0–1]^16^	0.86 ± 0.08 [0–1]^20^	0.80 ± 0.16 [0–1]^29^	0.98 ± 0.16 [0–1]^37^	0.81 ± 0.10 [0–1]^40^	0.74 ± 0.27 [0–1] ^40^
Non-renal clearance (mL/min)		24.6 ± 19.4 [0-66.8]^14–16^	20.8 ± 7 [10.1–37.7] ^18–21^	89.2 ± 31.9 [27.1–160.0]^24–28,30^	38.3 ± 25.6 [0-104.8]^32,34,36^	45.7 ± 38.3 [0-192.0]^38,40,41^	38.3 ± 66.2 [0-381.0] ^38–40^
SA	Qd = 300 ml/min	0.45 ± 0.09 [0–1]^14–17,42−44^	0.34 ± 0.06 [0–1] ^18–21,45^	0.34 ± 0.07 [0–1]^22–28,30,46–48^	0.37 ± 0.07 [0–1]^31–36,49−54^	0.31 ± 0.06 [0–1]^38,41,55–63^	0.33 ± 0.07 [0–1]^38,55–57,61,63,64^
	Qd = 100 ml/min	0.68 ± 0.14 [0–1] ^14–17,42−44^	0.58 ± 0.11 [0–1] ^18–21,45^	0.63 ± 0.12 [0–1] ^22–28,30,46–48^	0.65 ± 0.13 [0–1] ^31–36,49−54^	0.46 ± 0.09 [0–1]^38,41,55–63^	0.51 ± 0.10 [0–1] ^38,55–57,61,63,64^
	Qd = 50 ml/min	0.75 ± 0.15 [0–1] ^14–17,42−44^	0.73 ± 0.15 [0–1] ^18–21,45^	0.82 ± 0.16 [0–1] ^22–28,30,46–48^	0.83 ± 0.16 [0–1] ^31–36,49−54^	0.55 ± 0.11 [0–1] ^38,41,55–63^	0.63 ± 0.13 [0–1]^38,55–57,61,63,64^
SC	Quf = 5 ml/min	0.82 ± 0.16 [0–1] ^14–17,42−44^	1.0 ± 0.20 [0–1] ^18–21,45^	0.82 ± 0.16 [0–1] ^22–28,30,46–48^	0.98 ± 0.2 [0–1] ^31–36,49−54^	0.87 ± 0.17 [0–1] ^38,41,55–63^	1.0 ± 0.20 [0–1] ^38,55–57,61,63,64^

SA: saturation coefficient, SC: sieving coefficient, Qd = dialysate flow rate, Quf = ultrafiltrate flow rate. All values are expressed as mean ± standard deviation [range]

CL_HD_ = SA x Qd.

CL_UF_ = SC x Quf.

Ke_on = (CL_NR_ + CL_HD or UF_)/Vd (intra-KRT period).

Ke_off = CL_NR_ /Vd (inter-KRT period).

Where CL_HD_ is the transmembrane clearance during HHD, SA is the saturation coefficient, SC is the sieving coefficient, Qd is the dialysate flow rate, Quf is the ultrafiltrate flow rate, Ke_on is the elimination rate constant during KRT, CL_NR_ is non-renal clearance, Vd is volume of distribution, and Ke_off is the elimination rate constant between KRT treatments.

### Pharmacodynamic & safety targets

The pharmacodynamic (PD) targets for study drugs were free plasma drug concentration time above the minimum inhibitory concentration (fT > MIC) of the pathogen for 40%, 50% and 60% of the dosing interval (40%, 50%, and 60% fT > _MIC_) for carbapenems (imipenem and meropenem), penicillin (piperacillin) and cephalosporins (cefepime and ceftazidime) respectively [64, 65]. Furthermore, the attainment of the free plasma drug concentrations exceeding four times MIC (fT > _MICx4_) is associated with maximal bacterial killing effect of β-lactams [66, 67]. Attaining this latter aggressive PD target has been recommended for critically ill patients to optimize clinical efficacy while preventing bacterial resistance [68]. Thus, we used each of these two PD targets (40%, 50% or 60% fT_> MIC_ and 40%, 50% or 60% fT_> MICx4_) to predict the optimal drug doses in critically ill patients receiving KRT. For tazobactam, the target was to attain 50% free plasma drug concentration above the threshold concentration (50% fT > threshold) [69, 70]. Clinical and Laboratory Standards Institute susceptibility breakpoint MICs reported against the reference microorganism, *Pseudomonas aeruginosa*, were used to evaluate the probability of target attainment (PTA) of each dosing regimen: 2 mg/L for imipenem and meropenem, 16 mg/L for piperacillin with threshold tazobactam concentrations of 4 mg/L, and 8 mg/L for cefepime and ceftazidime [69].

In general, β-lactam antibiotics are considered safe and the dosing regimens are primarily determined by pharmacokinetic/pharmacodynamic target attainment. However, β-lactam-associated neurotoxicity has been correlated with high plasma concentrations and is more commonly reported in critically ill patients with kidney dysfunction [71–76]. Total plasma concentrations of > 64 mg/L and > 20 mg/L have been associated with increased neurotoxicity risk for meropenem and cefepime respectively [73–75]. A recent retrospective study suggested that the total piperacillin trough plasma concentrations of 157 mg/L in combination with tazobactam was linked to the incidence of neurologic disorders in critically ill patients [76]. No ceftazidime or imipenem studies have evaluated the relationship between neurotoxicity and plasma concentrations. However, it is suggested that free plasma concentrations should not exceed eight times the MIC for β-lactam antibiotics without validated toxicity threshold concentrations to lower the risk of toxicity [68]. We assessed the potential risk of neurotoxicity associated with each simulated drug dosing regimen by evaluating total plasma concentrations at the end of each day during one week of antibiotic therapy, using these suggested toxicity threshold concentrations (i.e. 20 mg/L, 64 mg/L, 157 mg/L, 16 mg/L, and 64 mg/L for cefepime, ceftazidime, piperacillin, imipenem and meropenem, respectively).

### Monte Carlo simulation and optimal dosing regimen

Various dosing regimens of each study drug were simulated using 0.5-hour intermittent infusions. Additionally, 4-hour extended infusions were simulated for cefepime and piperacillin/tazobactam. In KRT settings 1–3, the initial doses were infused immediately after a 4-hour HD. For KRT setting 4 with daily 9-hour PIKRT, each drug dosing regimen was infused in the 2 scenarios representing the possible extreme infusion situations: (1) “Early PIKRT” where the initial dose is infused at the beginning of PIKRT and (2) “Late PIKRT” where the initial dose is given 15 h prior to PIKRT. Monte Carlo simulation (MCS) [Crystal Ball Classroom Edition, Oracle] generated a week of free plasma drug concentration-time profiles of each simulated dosing regimen in 5,000 virtual patients in each of 5 KRT settings. Then, the PTA of each simulated dosing regimen was calculated for the different PD targets. A dosing regimen was considered optimal if PTA was attained in ≥ 90% of 5,000 virtual patients with the smallest daily dose during one week of antibiotic therapy.

## Results

MCS determined the optimal dosing regimens for all study antibiotics in all five KRT settings (Table 2). It is predicted that alterations in KRT regimens would influence on the PTA of dosing regimens, necessitating different antibiotic doses to attain desired efficacy targets. It should be noted that the clinical relevance and potential for toxicity were considered when selecting optimal doses for aggressive PD targets (40–60% fT_> MICx4_). Thus, when antibiotic dose regimens yielded a PTA slightly below 90%, particularly on day 1, but considered clinically acceptable, these regimens were deemed optimal. Furthermore, if achieving PTA of $\ge$90% necessitated a dose greater than the maximal conventional dose and/or substantially increased the risk of potential toxicity, antibiotic regimens with a PTA less than 90% were also accepted as optimal following a careful evaluation of benefits vs. risks. Overall, the recommended doses for thrice-weekly HD, daily HD, and sequential therapy (KRT settings 1–3) were consistent for all study antibiotics, while higher doses were needed for daily PIKRT therapy (settings 4 and 5). The PTAs of all simulated antibiotic doses in all five KRT settings were provided in supplementary materials.

**Table 2:** Five β-lactam dosing recommendations in five modeled kidney replacement therapies

Antibiotic agent	PD target†	Dosing Recommendation in Five KRTs				
		1. Thrice-weekly HD^1^	2. Daily HD^1^	3. Sequential Therapy^2^	4. 9-hour PIKRT^3^	5. Extended PIKRT^4^
Cefepime	60% fT_> MIC_	2 g LD, 1 g q24h post-HD			1 g q12h	2 g LD, 1 g q12h
	60% fT_> MICx4_	3 g LD, 2 g q12h post-HD			3 g LD, 1 g q6h	2 g q8h
Ceftazidime	60% fT_> MIC_	1 g q24h post-HD			1 g q12h	1 g q12h
	60% fT_> MICx4_	2 g LD, 1 g q8h post-HD			2 g LD, 1 g q8h	2 g q8h
Imipenem	40% fT_> MIC_	500 mg q12h post-HD			500 mg q12h	500 mg q8h
	40% fT_> MICx4_	750 mg q8h post-HD			1 g q8h	750 mg q6h
Meropenem	40% fT_> MIC_	500 mg q24h post-HD			500 mg q12h	500 mg q12h
	40% fT_> MICx4_	1 g LD, 500 mg q12h post-HD			500 mg q8h	1 g q12h
Piperacillin/ tazobactam	50% fT_> MIC_	4.5 g q12h post-HD			4.5 g q12h	3.375 g q8h
	50% fT_> MICx4_	4.5 g q8h post-HD or 3.375 g q6h post-HD			4.5 g q8h or 3.375 g q6h	4.5 g q6h

^1^HD: 4-hour hemodialysis with dialysate flow rate (Qd) 300 ml/min; ^2^Sequential therapy: 4-hour hemodialysis with Qd 300 ml/min, then 20-hour ultrafiltration at 5 ml/min; ^3^PIKRT: prolonged intermittent kidney replacement therapy at Qd 100 ml/min; ^4^Extended PIKRT: 24-hour hemodialysis at Qd 50 ml/min

PD: pharmacodynamic; LD: loading dose

†40%,50% and 60% fT_> MIC_ or fT_> MICx4_ denotes at least 40%, 50%, and 60% of time during each day of one week of antibiotic therapy that the free plasma drug concentration was greater than the target minimum inhibitory concentration (MIC) or four time of the target MIC; Piperacillin doses are the ones that attain both piperacillin efficacy targets and tazobactam efficacy target (i.e. 50% fT > threshold concentration of 4 mg/L); Susceptibility breakpoint MIC for *P. aeruginosa* was used (i.e. 8 mg/L for cefepime and ceftazidime, 2 mg/L for imipenem and meropenem, and 16 mg/L for piperacillin)

Doses are ones attaining efficacy target of either 40%,50% and 60% fT_> MIC_ or fT_> MICx4_ in ∼90% of 5,000 virtual patients receiving each of 5 kidney replacement therapies; All doses are started after a HD session in settings 1–3. All doses above are given as a 0.5-hour intermittent infusion except piperacillin/tazobactam which may be administered as either 0.5-hour intermittent infusion or 4-hour extended infusion

Notably, the 9-hour PIKRT setting (KRT setting 4) presented a challenge in that two extreme dosing scenarios could potentially exist contingent upon the timing of antibiotic administration relative to the PIKRT. Antibiotic initial doses given at the initiation of 9-hour PIKRT (early PIKRT) yielded different antibiotic exposures compared to those infusions occurring 15 h before the 9-hour PIKRT (late PIKRT). However, our recommended antibiotic doses for 9-hour PIKRT setting achieved PTA ∼90% independent of when the antibiotic was infused in relation to PIKRT.

MCS also evaluated the safety of each antibiotic dosing regimen utilizing the suggested safety threshold from the literature. Overall, the potential for a substantial increase in neurotoxicity risk were more pronounced with the MCS-recommended cefepime and piperacillin/tazobactam dosing regimens designed to attain aggressive PD targets. Tables 3 and 4 present the probability of increased neurotoxicity risk with MCS-recommended cefepime and piperacillin/tazobactam dosing regimens respectively in all five KRT settings. The recommended ceftazidime dose for aggressive PD target (60% fT_> MICx4_) also elevated drug concentrations above the safety threshold in 25–52% of simulated patients in thrice-weekly HD (KRT setting 1). Additionally, the recommended imipenem doses for aggressive PD target (40% fT_> MICx4_) resulted in drug concentrations exceeding the safety threshold in 0.3–8.3% and 9.0-11.9% of the virtual cohort in thrice-weekly HD and Early 9-hour PIKRT settings, respectively. Further information on the potential neurotoxicity risk predicted with MCS-recommended dosing regimens for ceftazidime, imipenem and meropenem are reported in supplementary materials.

**Table 3:** Probability of neurotoxicity of MCS-driven cefepime dosing recommendation in five kidney replacement therapies

	KRT setting	PD target^†^	MCS-driven Cefepime dosing recommendation	Probability of total concentration above neurotoxicity threshold at the end of each day during 1 week of therapy^£^						
				Day 1	Day 2	Day 3	Day 4	Day 5	Day 6	Day 7
1	4-hour HD on Mon-Wed-Fri	60% fT > MIC	2 g LD, 1 g q24h post-HD	58.5%	10.0%	41.9%	10.7%	41.2%	51.9%	16.6%
		60% fT > MICx4	3 g LD, 2 g q12h post-HD	98.2%	84.7%	98.3%	85.1%	98.3%	98.4%	85.5%
2	4-hour HD daily	60% fT > MIC	2 g LD, 1 g q24h post-HD	1.9%	1.1%	1.1%	1.2%	1.3%	1.5%	1.5%
		60% fT > MICx4	3 g LD, 2 g q12h post-HD	62.9%	70.0%	70.6%	70.6%	70.6%	70.6%	70.6%
3	Sequential 4-hour HD & 20-hour UF	60% fT > MIC	2 g LD, 1 g q24h post-HD	0.7%	0.3%	0.3%	0.3%	0.4%	0.4%	0.4%
		60% fT > MICx4	3 g LD, 2 g q12h post-HD	58.4%	65.7%	66.2%	66.3%	66.3%	66.3%	66.3%
4	Early 9-hour PIKRT^€^ daily	60% fT > MIC	1 g q12h	58.1%	74.7%	77.4%	78.0%	78.2%	78.2%	78.2%
		60% fT > MICx4	2 g LD, 1 g q6h	99.9%	99.9%	99.9%	99.9%	99.9%	99.9%	99.9%
	Late 9-hour PIKRT^€^ daily	60% fT > MIC	1 g q12h	1.6%	16.4%	23.2%	25.0%	25.6%	25.7%	25.8%
		60% fT > MICx4	2 g LD, 1 g q6h	81.6%	80.3%	80.3%	81.3%	80.3%	80.3%	80.3%
5	Extended PIKRT daily	60% fT > MIC	1 g q12h	2.1%	11.8%	16.4%	17.7%	18.1%	18.3%	18.4%
		60% fT > MICx4	2 g q8h	62.2%	67.9%	68.2%	68.2%	68.2%	68.2%	68.2%

KRT: kidney replacement therapy; PD: pharmacodynamic; MCS: Monte Carlo simulation; HD: Hemodialysis at dialysate flow rate (Qd) 300 ml/min; UF: Ultrafiltration at ultrafiltration flow rate of 5 ml/min; PIKRT: Prolonged intermittent kidney replacement therapy (9-hour PIKRT runs at Qd 100 ml/minl and extended PIKRT runs at Qd 50 ml/min for 24 h); LD: loading dose

^†^60% fT_> MIC_ or 60% fT_> MICx4_ denotes at least 60% of time during each day of one week of antibiotic therapy that the free plasma drug concentration was greater than the target minimum inhibitory concentration (MIC) or four time of the target MIC of 8 mg/L (susceptibility breakpoint MIC for *P. aeruginosa*)

^£^This indicates the percentage of 5000 simulated patients that were at or above the suggested cefepime neurotoxicity threshold (i.e. total plasma concentration ≥ 20 mg/L) at the end of each day during 1 week of therapy

^€^ EARLY PIKRT is where the initial cefepime dose is infused at the beginning of 9-hour PIKRT and late PIKRT where the initial cefepime dose is given 15 h prior to 9-hour PIKRT

**Table 4:** Probability of neurotoxicity of MCS-driven piperacillin/tazobactam dosing recommendation in five kidney replacement therapies

	KRT setting	PD target^†^	MCS-driven Piperacillin/tazobactam dosing recommendation	Probability of total concentration above neurotoxicity threshold at the end of each day during 1 week of therapy^£^						
				Day 1	Day 2	Day 3	Day 4	Day 5	Day 6	Day 7
1	4-hour HD on Mon-Wed-Fri	50% fT > MIC	4.5 g q12h post-HD	10.9%	2.0%	22.6%	6.5%	24.7%	29.9%	10.1%
		50% fT > MICx4	4.5 g q8h post-HD	36.8%	15.7%	50.3%	22.5%	51.2%	54.9%	24.9%
			3.375 g q6h post-HD	41.0%	16.8%	53.5%	22.6%	54.3%	57.8%	25.5%
2	4-hour HD daily	50% fT > MIC	4.5 g q12h post-HD	0.0%	0.3%	1.1%	1.7%	2.3%	2.4%	2.6%
		50% fT > MICx4	4.5 g q8h post-HD	1.3%	9.5%	13.4%	14.6%	15.1%	15.2%	15.3%
			3.375 g q6h post-HD	1.5%	9.8%	14.1%	15.8%	16.4%	16.6%	16.7%
3	Sequential 4-hour HD & 20-hour UF	50% fT > MIC	4.5 g q12h post-HD	0.0%	0.0%	0.5%	1.0%	1.2%	1.4%	1.6%
		50% fT > MICx4	4.5 g q8h post-HD	0.4%	5.7%	9.2%	10.4%	10.0%	10.7%	11.0%
			3.375 g q6h post-HD	0.7%	6.6%	10.2%	11.4%	11.9%	12.0%	12.1%
4	Early 9-hour PIKRT^€^ daily	50% fT > MIC	4.5 g q12h	3.1%	7.8%	10.5%	11.7%	12.1%	12.3%	12.5%
		50% fT > MICx4	4.5 g q8h	28.6%	40.1%	43.4%	44.2%	44.6%	44.7%	44.8%
			3.375 g q6h	45.8%	59.1%	61.5%	62.4%	62.6%	62.7%	62.7%
	Late 9-hour PIKRT^€^ daily	50% fT > MIC	4.5 g q12h	0.0%	0.2%	0.6%	1.0%	1.4%	1.7%	1.8%
		50% fT > MICx4	4.5 g q8h	1.1%	9.1%	13.2%	14.7%	15.1%	15.0%	15.1%
			3.375 g q6h	1.7%	9.8%	14.0%	15.6%	15.6%	15.7%	15.7%
5	Extended PIKRT daily	50% fT > MIC	3 g q8h	0.0%	0.3%	0.8%	1.1%	1.2%	1.3%	1.4%
		50% fT > MICx4	4 g q6h	17.4%	28.9%	31.1%	31.5%	31.7%	31.8%	31.8%

KRT: kidney replacement therapy; PD: pharmacodynamic; MCS: Monte Carlo simulation; HD: Hemodialysis at dialysate flow rate (Qd) 300 ml/min; UF: Ultrafiltration at ultrafiltration flow rate of 5 ml/min; PIKRT: Prolonged intermittent kidney replacement therapy (9-hour PIKRT runs at Qd 100 ml/minl and extended PIKRT runs at Qd 50 ml/min for 24 h); LD: loading dose

^†^50% fT_> MIC_ or 50% fT_> MICx4_ denotes at least 50% of time during each day of one week of antibiotic therapy that the free plasma piperacillin concentration was greater than the target minimum inhibitory concentration (MIC) or four time of the target MIC of 16 mg/L (susceptibility breakpoint MIC for *P. aeruginosa*)

^£^This indicates the percentage of 5000 simulated patients that were at or above the suggested piperacillin safety threshold (i.e. free plasma concentration ≥ 157 mg/L) at the end of each day during 1 week of therapy

^€^ EARLY PIKRT is where the initial piperacillin/tazobactam dose is infused at the beginning of 9-hour PIKRT and late PIKRT where the initial piperacillin/tazobactam dose is given 15 h prior to 9-hour PIKRT

Interestingly, a 4-hour extended infusion strategy, commonly utilized in clinical practice, did not appreciably enhance PTA compared to 0.5-hour intermittent infusion, and did not alter the selection of optimal cefepime and piperacillin/tazobactam doses in our analyses (supplementary materials)

## Discussion

This is the first study to attempt to develop common antibiotic dosing recommendations using MCS for the breadth of KRT options available for critically ill patients receiving newer KRT. MCS was able to identify plausible antibiotic doses in all five KRT settings that would attain PD targets. As aforementioned, the same antibiotic doses were recommended for KRT settings 1–3 [i.e. thrice-weekly HD, daily HD, and sequential therapy (4-hour HD, followed by 20-hour UF daily)]. However, a higher proportion of simulated patients exhibited an increased risk of drug-induced neurotoxicity in the thrice-weekly HD setting compared to daily HD or sequential therapy, attributed to less frequent HD sessions per week. The recommended antibiotic doses to attain less aggressive PD targets (40–60% fT_> MIC_) in these KRT settings 1–3 were similar to those recommended for end stage kidney disease patients with HD [77–81], while higher antibiotic doses were necessary to attain aggressive PD targets (40–60% fT_> MICx4_). For KRT settings 4 and 5 [i.e. 9-hour and extended PIKRT], the recommended antibiotic doses were 50–100% higher than those for KRT settings 1–3, except piperacillin/tazobactam.

The MCS analysis predicted that piperacillin/tazobactam 4.5 g q12h, and 4.5 g q8h or 3.375 g q6h would attain piperacillin PD targets of 50% fT_> MIC_ and 50% fT_> MICx4_ respectively, while concurrently achieving the tazobactam target of 50% fT > threshold in KRT settings 1–4. It should be noted that these drug dosing regimens should be administered post-HD in KRT settings 1–3 but can be given regardless of the timing of 9-hour PIKRT in setting 4. The consistent piperacillin/tazobactam doses recommended for KRT settings 1–4 are likely due to preserved and robust piperacillin non-renal clearance observed in patients with AKI receiving KRT [38, 40, 41] and similar total piperacillin dialytic clearance during HD and 9-hour PIKRT. Piperacillin non-renal clearance is substantial (45.7 + 38.3 ml/min), and the frequency of KRT did not significantly influence PTA in simulated patients. Moreover, the estimated piperacillin extracorporeal clearances during HD with Qd 300 ml/min and 9-hour PIKRT with Qd 100 ml/min were ∼93 ml/min and ∼46 ml/min respectively. Consequently, total piperacillin removal during a 4-hour HD or a 9-hour PIKRT was comparable, resulting in similar PTA and the selection of the same optimal piperacillin dosing regimen in KRT settings 1–4. It should be also noted that for less aggressive PD target (50% fT_> MIC_), smaller piperacillin doses (i.e. 2 g q12h and 3 g q12h) than recommended (i.e. 4 g q12h) would attain 90% PTA in these KRT settings but the accompanying tazobactam doses (i.e. 0.25 g q12h and 0.375 g q12h) did not successfully attain the target of 50% fT > threshold. Attainment of acceptable PTA for 50% fT > threshold required tazobactam 0.5 g q12h. Thus, piperacillin/tazobactam 4.5 g q12h was chosen as the optimal regimen to meet both piperacillin and tazobactam targets in these KRT settings.

Extended infusion is a common strategy to maximize the time-dependent bactericidal activity of β-lactam antibiotics. In our MCS study, we evaluated the PTA of cefepime and piperacillin/tazobactam doses with extended infusion in critically ill patients receiving KRT. Administering these antibiotics with a 4-hour extended infusion resulted in a slight increase in PTA by 0–4% for cefepime and by 1–5% for piperacillin, respectively, compared to the same doses given via a 0.5-hour intermittent infusion. However, this PTA increase did not affect the selection of optimal cefepime and piperacillin/tazobactam doses in our analysis. Extended infusion was advantageous in achieving a PTA of ≥ 90% when cefepime and piperacillin doses with intermittent infusion yielded slightly below 90% PTA. For example, in KRT setting 2, piperacillin 4 g q8h with intermittent infusion resulted PTA 88–89%, but when administered with extended infusion, the PTA increased to 91–92%. Nevertheless, these PTA differences between intermittent vs. extended infusion did not appear clinically significant in our simulated patients.

β-lactam antibiotics are generally considered safe; however, in recent years, there has been an increasing recognition of neurological deterioration in critically ill patients receiving a β-lactam [72, 82]. In this MCS analysis, we also assessed the safety of each antibiotic dosing regimen at the end of each day over a 1-week treatment period and found a substantial increase in the risk of neurotoxicity with the recommended cefepime and piperacillin/tazobactam doses, particularly to attain the aggressive PD targets (Tables 3 and 4). Notably, the cefepime safety threshold used in our analysis (i.e., total trough concentration of ≥ 20 mg/L) would be approximately equivalent to a free drug concentration of 16 mg/L, assuming protein binding of 20% [16]. This safety threshold (16 mg/L) closely approaches the PD target threshold concentrations (MIC of 8 mg/L or MICx4 of 32 mg/L). Unavoidably, cefepime doses attaining the aggressive PD target yielded total cefepime concentration exceeding the safety threshold (16 mg/L) at the end of each simulated day in many simulated patients (58–99%). The MCS also indicated that piperacillin/tazobactam doses achieving the aggressive PD target would elevate the risk of neurotoxicity in up to 63% of virtual cohort. For safety reasons, we deemed cefepime and piperacillin/tazobactam doses slight below 90% of the PTA as optimal, if higher doses substantially increased the risk of neurotoxicity. Furthermore, we accepted the doses that resulted in PTAs of less than 80% on day 1, but consistently attained PTAs ∼90% for the remainder of the week. For instance, in early 9-hour PIKRT and extended PIKRT settings, cefepime 3 g LD then 1 g q6h and 2 g q8h yielded PTAs of 77% and 63% respectively on day 1 while maintaining PTA ∼90% for the rest of week. The MCS predicted that PTA ≥ 90% on day 1 to attain aggressive PD target in these KRT settings would require cefepime doses of up to 8 g/day exceeding the maximal conventional daily dose (6 g/day). Thus, we selected these cefepime doses as optimal not to exceed 6 g/day despite the lower PTA on day 1. With similar considerations, we accepted piperacillin/tazobactam doses as optimal for aggressive PD target in KRT settings 2–4, even though they resulted in PTAs of less than 80% on day 1, as they achieved PTA ∼90% for the rest of the week. When a clinician seeks to ensure target attainment on day 1, a higher cefepime or piperacillin/tazobactam LD may be prescribed on day 1, after evaluating the benefits vs. toxicity risk based on the MCS results provided in this report.

Clinicians should be vigilant about the potential risk of neurotoxicity with the recommended cefepime and piperacillin/tazobactam doses in simulated KRT settings. They also should practice careful monitoring to detect any clinical manifestations of neurotoxicity. If available, therapeutic drug monitoring (TDM) should be performed to optimize antibiotic therapy. Currently, β-lactam TDM is more commonly utilized in hospitals in some European countries and Australia but has been limited to research purposes in the U.S. and other regions [83, 84]. In clinical settings where β-lactam TDM is not readily accessible, clinicians should carefully weigh the benefits and risks based on the PTA and the potential neurotoxicity risk predicted in our MCS analyses. For patients at a high risk of neurotoxicity, clinicians should consider using cefepime and piperacillin/tazobactam doses designed to attain less aggressive PD targets or an alternative antibiotic.

This study has several limitations. First, we modelled the virtual patients based on published body weight and pharmacokinetic parameters derived from critically ill patients undergoing KRTs. We assumed that these virtual patients were anuric adults receiving uninterrupted KRT sessions. Additionally, our modeling was limited to only 5 KRT regimens. Therefore, the applicability of our MCS findings is confined to individuals matching to the modeled patient characteristics and receiving one of the modeled KRT settings. All the studied β-lactam antibiotics are primarily eliminated via the kidneys and are readily dialyzable. Consequently, patients with residual or improving kidney function might require higher doses than recommended, while prolonged interruptions in KRT may necessitate lower doses. Secondly, our study assumed a serious infection with *P. aeruginosa* in determining the initial β-lactam dosing recommendations. The susceptibility MIC breakpoints for *P. aeruginosa* are typically higher than those for other *Enterobacterales* [69]. If a different pathogen with lower MICs (e.g. <8 mg/L for cefepime and ceftazidime; <16 mg/L for piperacillin/tazobactam; <2 mg/L for imipenem and meropenem) is identified as the cause of infection, clinicians may consider reducing the initial dosing regimens based on the susceptibility results. Lastly, this study predicts that cefepime and piperacillin/tazobactam doses aimed to attain aggressive PD targets would result in plasma drug concentrations exceeding safety thresholds, thereby increasing the risk of neurotoxicity. It is strongly advisable that clinicians carefully assess the benefits and risks predicted with these regimens when considering treatment for this vulnerable patient population.

## Conclusions

Innovative KRT systems allow clinicians wider KRT flexibility than ever before. MCS was able to predict dosing recommendations for five commonly used β-lactam antibiotics for critically ill patients receiving wide variations in KRT applications. Vigilant monitoring for antibiotic adverse effects when attempting to attain aggressive PD targets is essential, especially for cefepime and piperacillin/tazobactam.

### Electronic supplementary material

Below is the link to the electronic supplementary material.

Supplementary Material 1

## Data Availability

The datasets used and/or analyzed during the current study are available from the corresponding author on reasonable request.

## Abbreviations

AKI: acute kidney injury
fT > _MIC_: time of free-serum concentration above the minimum inhibitory concentration
HD: hemodialysis
KRT: kidney replacement therapy
MCS: Monte Carlo Simulations
PIKRT: prolonged intermittent kidney replacement therapy
PK: pharmacokinetic
PD: pharmacodynamic
PTA: probability of target attainment
TDM: therapeutic drug monitoring
