# Supplementary material for: Antibiotic dosing recommendations in critically ill patients receiving new innovative kidney replacement therapy
Source: BMC Nephrol. 2024 Feb 27;25:73. doi: 10.1186/s12882-024-03469-2 (PMC10900833; doi:10.1186/s12882-024-03469-2)
Supplement: Supplementary file 1 — Supplementary Material 1 [file 12882_2024_3469_MOESM1_ESM.docx]

## Cefepime simulation results

**Table S1. PTA of various cefepime dosing regimens in KRT setting 1 (Thrice-weekly HD)**

| **Cefepime Dose** | **MIC**  **(mg/L)** | **PTA during 1-week of therapy** | | | | | | | |
| --- | --- | --- | --- | --- | --- | --- | --- | --- | --- |
|  |  | **Mean** | **Day 1** | **Day 2** | **Day 3** | **Day 4** | **Day 5** | **Day 6** | **Day 7** |
| 1g q24h post-HD | 8 | 91.3% | 80.9% | 91.9% | 90.7% | 92.4% | 90.9% | 90.9% | 92.5% |
|  | 32 | 90.8% | 1.5% | 12.2% | 5.7% | 19.2% | 7.5% | 7.5% | 21.7% |
| 2g LD, 1g q24h post-HD | 8 | 93.3% | 98.2% | 93.7% | 91.1% | 92.3% | 90.9% | 90.9% | 92.2% |
|  | 32 | 9.5% | 28.2% | 29.5% | 9.8% | 23.5% | 9.3% | 9.3% | 23.3% |
| 1g q12h post-HD | 8 | 99.9% | 96.2% | 100.0% | 100.0% | 100.0% | 100.0% | 100.0% | 100.0% |
|  | 32 | 61.6% | 10.6% | 55.7% | 53.9% | 71.0% | 61.2% | 61.2% | 73.4% |
| 2g q12h post-HD | 8 | 100.0% | 100.0% | 100.0% | 100.0% | 100.0% | 100.0% | 100.0% | 100.0% |
|  | 32 | 95.4% | 60.9% | 95.3% | 94.5% | 96.6% | 94.8% | 94.8% | 96.7% |
| 2g LD, 2g q12h EI post-HD | 8 | 100.0% | 100.0% | 100.0% | 100.0% | 100.0% | 100.0% | 100.0% | 100.0% |
|  | 32 | 95.4% | 60.8% | 95.4% | 95.6% | 97.7% | 95.9% | 95.9% | 97.9% |
| 3g LD, 2g q12h post-HD | 8 | 100.0% | 100.0% | 100.0% | 100.0% | 100.0% | 100.0% | 100.0% | 100.0% |
|  | 32 | 96.1% | 86.0% | 96.0% | 95.1% | 96.7% | 95.2% | 95.2% | 96.7% |
| 3g LD, 2g q12h EI post-HD | 8 | 100.0% | 100.0% | 100.0% | 100.0% | 100.0% | 100.0% | 100.0% | 100.0% |
|  | 32 | 97.2% | 85.6% | 97.2% | 96.2% | 97.9% | 96.3% | 96.3% | 98.1% |
| 3g q12h on day 1, then 2g q12h post-HD | 8 | 100.0% | 100.0% | 100.0% | 100.0% | 100.0% | 100.0% | 100.0% | 100.0% |
|  | 32 | 97.1% | 89.0% | 97.5% | 95.3% | 97.0% | 95.1% | 95.1% | 97.0% |
| 2g q8h on day 1, then 2g q12h post-HD | 8 | 100.0% | 100.0% | 100.0% | 100.0% | 100.0% | 100.0% | 100.0% | 100.0% |
|  | 32 | 96.8% | 92.5% | 98.1% | 94.9% | 96.4% | 94.7% | 94.7% | 96.4% |
| 1g q8h post-HD | 8 | 99.9% | 99.9% | 99.9% | 99.9% | 99.9% | 99.9% | 99.9% | 99.9% |
|  | 32 | 69.2% | 42.4% | 74.5% | 60.9% | 74.2% | 62.6% | 62.6% | 75.0% |
| 2g q8h post-HD | 8 | 100.0% | 99.9% | 100.0% | 100.0% | 100.0% | 100.0% | 100.0% | 100.0% |
|  | 32 | 97.1% | 92.5% | 98.1% | 95.1% | 96.8% | 94.9% | 94.9% | 96.8% |

MIC: minimum inhibitory concentration; PTA: probability of target attainment; 60%*f*T> MIC: 60% of a dosing interval that a free drug concentration exceeds the MIC.

EI: extended infusion over 4 hours. All other doses were simulated as a 30 min infusion.

The dose in yellow and green is the smallest cefepime dosing regimen attaining PD targets of 60% fT>MIC of 8 mg/L and 60% fT>MICx4 of 32mg/L respectively.

**Table S2. PTA of various cefepime dosing regimens in KRT setting 2 (Daily HD)**

| **Cefepime Dose** | **MIC**  **(mg/L)** | **PTA during 1-week of therapy** | | | | | | | |
| --- | --- | --- | --- | --- | --- | --- | --- | --- | --- |
|  |  | **Mean** | **Day 1** | **Day 2** | **Day 3** | **Day 4** | **Day 5** | **Day 6** | **Day 7** |
| 1g q24h post-HD | 8 | 89.8% | 80.3% | 89.5% | 90.4% | 90.5% | 90.6% | 90.6% | 90.6% |
|  | 32 | 3.1% | 1.4% | 3.0% | 3.5% | 3.8% | 3.9% | 3.9% | 3.9% |
| 2g LD, 1g q24h post-HD | 8 | 92.0% | 98.7% | 91.7% | 90.4% | 90.3% | 90.3% | 90.3% | 90.2% |
|  | 32 | 4.8% | 28.2% | 6.7% | 4.4% | 4.1% | 4.0% | 4.0% | 4.0% |
| 1g q12h post-HD | 8 | 99.9% | 95.4% | 99.9% | 99.9% | 99.9% | 99.9% | 99.9% | 99.9% |
|  | 32 | 30.4% | 7.6% | 24.2% | 31.8% | 35.0% | 36.5% | 36.5% | 37.4% |
| 2g q12h post-HD | 8 | 100.0% | 99.9% | 100.0% | 100.0% | 100.0% | 100.0% | 100.0% | 100.0% |
|  | 32 | 90.8% | 56.4% | 88.3% | 91.9% | 92.4% | 92.5% | 92.5% | 92.5% |
| 2g LD, 2g q12h EI post-HD | 8 | 100.0% | 100.0% | 100.0% | 100.0% | 100.0% | 100.0% | 100.0% | 100.0% |
|  | 32 | 90.7% | 57.0% | 88.0% | 92.0% | 92.7% | 92.8% | 92.8% | 92.9% |
| 3g LD, 2g q12h post-HD | 8 | 100.0% | 100.0% | 100.0% | 100.0% | 100.0% | 100.0% | 100.0% | 100.0% |
|  | 32 | 93.6% | 84.8% | 92.2% | 93.4% | 93.6% | 93.7% | 93.7% | 93.7% |
| 3g LD, 2g q12h EI post-HD | 8 | 100.0% | 100.0% | 100.0% | 100.0% | 100.0% | 100.0% | 100.0% | 100.0% |
|  | 32 | 91.5% | 82.9% | 89.9% | 91.5% | 91.8% | 91.9% | 91.9% | 92.0% |
| 3g q12h on day 1, then 2g q12h post-HD | 8 | 100.0% | 100.0% | 100.0% | 100.0% | 100.0% | 100.0% | 100.0% | 100.0% |
|  | 32 | 94.0% | 86.4% | 94.2% | 93.5% | 93.4% | 93.4% | 93.4% | 93.4% |
| 2g q8h on day 1, then 2g q12h post-HD | 8 | 100.0% | 100.0% | 100.0% | 100.0% | 100.0% | 100.0% | 100.0% | 100.0% |
|  | 32 | 93.5% | 90.1% | 94.7% | 93.2% | 93.1% | 93.0% | 93.0% | 93.0% |
| 1g q8h post-HD | 8 | 100.0% | 100.0% | 100.0% | 100.0% | 100.0% | 100.0% | 100.0% | 100.0% |
|  | 32 | 70.3% | 22.1% | 59.8% | 69.8% | 73.6% | 75.5% | 75.5% | 75.1% |
| 2g q8h post-HD | 8 | 100.0% | 100.0% | 100.0% | 100.0% | 100.0% | 100.0% | 100.0% | 100.0% |
|  | 32 | 99.7% | 90.1% | 99.6% | 99.8% | 99.8% | 99.8% | 99.8% | 99.8% |

MIC: minimum inhibitory concentration; PTA: probability of target attainment; 60%*f*T> MIC: 60% of a dosing interval that a free drug concentration exceeds the MIC.

EI: extended infusion over 4 hours. All other doses were simulated as a 30 min infusion.

The dose in yellow and green is the smallest cefepime dosing regimen attaining PD targets of 60% fT>MIC of 8 mg/L and 60% fT>MICx4 of 32mg/L respectively.

**Table S3. PTA of various cefepime dosing regimens in KRT setting 3 (Sequential Therapy)**

| **Cefepime Dose** | **MIC**  **(mg/L)** | **PTA during 1-week of therapy** | | | | | | | |
| --- | --- | --- | --- | --- | --- | --- | --- | --- | --- |
|  |  | **Mean** | **Day 1** | **Day 2** | **Day 3** | **Day 4** | **Day 5** | **Day 6** | **Day 7** |
| 1g q24h post-HD | 8 | 85.2% | 75.1% | 85.0% | 86.1% | 86.3% | 86.4% | 86.4% | 86.4% |
|  | 32 | 0.8% | 0.3% | 0.8% | 1.0% | 1.0% | 1.0% | 1.0% | 1.0% |
| 2g LD, then 1g q24h post-HD | 8 | 89.4% | 98.3% | 89.1% | 87.8% | 87.5% | 87.4% | 87.4% | 87.4% |
|  | 32 | 1.5% | 20.4% | 2.3% | 1.2% | 1.0% | 0.9% | 0.9% | 0.9% |
| 2g LD, then 1g q24h EI post-HD | 8 | 91.1% | 97.8% | 90.7% | 89.5% | 89.3% | 89.3% | 89.3% | 89.3% |
|  | 32 | 1.1% | 19.8% | 1.8% | 0.9% | 0.8% | 0.7% | 0.7% | 0.7% |
| 1g q12h post-HD | 8 | 99.8% | 95.6% | 99.8% | 99.8% | 99.8% | 99.8% | 99.8% | 99.8% |
|  | 32 | 20.5% | 5.6% | 16.6% | 21.9% | 24.4% | 25.6% | 25.6% | 26.3% |
| 2g q12h post-HD | 8 | 100.0% | 100.0% | 100.0% | 100.0% | 100.0% | 100.0% | 100.0% | 100.0% |
|  | 32 | 88.9% | 53.2% | 86.2% | 90.5% | 91.3% | 91.4% | 91.4% | 91.5% |
| 2g LD, 2g q12h EI post-HD | 8 | 100.0% | 100.0% | 100.0% | 100.0% | 100.0% | 100.0% | 100.0% | 100.0% |
|  | 32 | 88.4% | 52.2% | 85.2% | 89.8% | 90.5% | 90.7% | 90.7% | 90.7% |
| 3g LD, 2g q12h post-HD | 8 | 100.0% | 100.0% | 100.0% | 100.0% | 100.0% | 100.0% | 100.0% | 100.0% |
|  | 32 | 90.9% | 81.8% | 89.2% | 90.6% | 90.9% | 91.0% | 91.0% | 91.0% |
| 3g LD, 2g q12h EI post-HD | 8 | 100.0% | 100.0% | 100.0% | 100.0% | 100.0% | 100.0% | 100.0% | 100.0% |
|  | 32 | 89.6% | 81.9% | 87.9% | 89.6% | 90.1% | 90.2% | 90.2% | 90.3% |
| 3g q12h on day 1, then 2g q12h post-HD | 8 | 100.0% | 100.0% | 100.0% | 100.0% | 100.0% | 100.0% | 100.0% | 100.0% |
|  | 32 | 91.3% | 85.8% | 91.9% | 90.9% | 90.7% | 90.7% | 90.7% | 90.7% |
| 2g q8h on day 1, then 2g q12h post-HD | 8 | 100.0% | 100.0% | 100.0% | 100.0% | 100.0% | 100.0% | 100.0% | 100.0% |
|  | 32 | 91.2% | 86.8% | 92.5% | 90.9% | 90.7% | 90.7% | 90.7% | 90.7% |
| 1g q8h post-HD | 8 | 100.0% | 99.9% | 100.0% | 100.0% | 100.0% | 100.0% | 100.0% | 100.0% |
|  | 32 | 62.5% | 18.4% | 53.2% | 62.8% | 67.1% | 67.8% | 67.8% | 68.4% |
| 2g q8h post-HD | 8 | 100.0% | 100.0% | 100.0% | 100.0% | 100.0% | 100.0% | 100.0% | 100.0% |
|  | 32 | 99.2% | 88.2% | 99.0% | 99.4% | 99.4% | 99.4% | 99.4% | 99.4% |

MIC: minimum inhibitory concentration; PTA: probability of target attainment; 60%*f*T> MIC: 60% of a dosing interval that a free drug concentration exceeds the MIC.

EI: extended infusion over 4 hours. All other doses were simulated as a 30 min infusion.

The dose in yellow and green is the smallest cefepime dosing regimen attaining PD targets of 60% fT>MIC of 8 mg/L and 60% fT>MICx4 of 32mg/L respectively.

**Table S4. PTA of various cefepime dosing regimens in KRT setting 4 (Early 9-hour PIKRT)**

| **Cefepime Dose** | **MIC**  **(mg/L)** | **PTA during 1-week of therapy** | | | | | | | |
| --- | --- | --- | --- | --- | --- | --- | --- | --- | --- |
|  |  | **Mean** | **Day 1** | **Day 2** | **Day 3** | **Day 4** | **Day 5** | **Day 6** | **Day 7** |
| 1g q24h | 8 | 27.1% | 4.0% | 21.8% | 27.8% | 29.6% | 30.0% | 30.0% | 30.2% |
|  | 32 | 0.0% | 0.0% | 0.0% | 0.0% | 0.0% | 0.0% | 0.0% | 0.0% |
| 2g LD, 1g q24h | 8 | 33.3% | 62.1% | 36.5% | 30.7% | 29.6% | 29.3% | 29.3% | 29.3% |
|  | 32 | 0.0% | 0.0% | 0.0% | 0.0% | 0.0% | 0.0% | 0.0% | 0.0% |
| 1g q12h | 8 | 98.7% | 91.9% | 98.8% | 98.8% | 98.8% | 98.8% | 98.8% | 98.8% |
|  | 32 | 10.7% | 0.3% | 11.7% | 15.8% | 18.0% | 19.0% | 19.0% | 19.3% |
| 2g q12h | 8 | 100.0% | 100.0% | 100.0% | 100.0% | 100.0% | 100.0% | 100.0% | 100.0% |
|  | 32 | 78.6% | 36.7% | 77.1% | 80.8% | 81.3% | 81.5% | 81.5% | 81.5% |
| 1g q8h | 8 | 100.0% | 99.9% | 100.0% | 100.0% | 100.0% | 100.0% | 100.0% | 100.0% |
|  | 32 | 53.5% | 9.6% | 44.5% | 55.2% | 58.7% | 59.5% | 59.5% | 59.9% |
| 2g q8h | 8 | 100.0% | 100.0% | 100.0% | 100.0% | 100.0% | 100.0% | 100.0% | 100.0% |
|  | 32 | 98.1% | 77.5% | 98.3% | 98.6% | 98.7% | 98.7% | 98.7% | 98.7% |
| 2g LD, 2g q8h EI | 8 | 100.0% | 100.0% | 100.0% | 100.0% | 100.0% | 100.0% | 100.0% | 100.0% |
|  | 32 | 99.5% | 65.2% | 99.1% | 99.6% | 99.7% | 99.7% | 99.7% | 99.7% |
| 3g LD, 2g q8h | 8 | 100.0% | 100.0% | 100.0% | 100.0% | 100.0% | 100.0% | 100.0% | 100.0% |
|  | 32 | 98.4% | 89.7% | 98.3% | 98.5% | 98.5% | 98.5% | 98.5% | 98.5% |
| 1g q6h | 8 | 100.0% | 99.9% | 100.0% | 100.0% | 100.0% | 100.0% | 100.0% | 100.0% |
|  | 32 | 83.1% | 13.8% | 79.7% | 85.3% | 86.2% | 86.4% | 86.4% | 86.3% |
| 1g LD, 1g q6h EI | 8 | 100.0% | 99.9% | 100.0% | 100.0% | 100.0% | 100.0% | 100.0% | 100.0% |
|  | 32 | 83.9% | 11.8% | 79.3% | 87.8% | 88.9% | 89.2% | 89.2% | 89.2% |
| 2g LD, 1g q6h | 8 | 100.0% | 100.0% | 100.0% | 100.0% | 100.0% | 100.0% | 100.0% | 100.0% |
|  | 32 | 83.3% | 52.2% | 81.3% | 84.5% | 85.1% | 85.3% | 85.3% | 85.4% |
| 2g LD, 1g q6h EI | 8 | 100.0% | 99.8% | 100.0% | 100.0% | 100.0% | 100.0% | 100.0% | 100.0% |
|  | 32 | 86.2% | 36.7% | 83.7% | 88.8% | 89.7% | 89.9% | 89.9% | 89.9% |
| 3g LD, 1g q6h | 8 | 100.0% | 99.8% | 100.0% | 100.0% | 100.0% | 100.0% | 100.0% | 100.0% |
|  | 32 | 85.9% | 76.6% | 84.6% | 86.1% | 86.4% | 86.5% | 86.5% | 86.5% |
| 3g LD, 1g q6h EI | 8 | 100.0% | 100.0% | 100.0% | 100.0% | 100.0% | 100.0% | 100.0% | 100.0% |
|  | 32 | 87.6% | 69.0% | 86.1% | 88.5% | 89.0% | 89.1% | 89.1% | 89.1% |

MIC: minimum inhibitory concentration; PTA: probability of target attainment; 60%*f*T> MIC: 60% of a dosing interval that a free drug concentration exceeds the MIC.

EI: extended infusion over 4 hours. All other doses were simulated as a 30 min infusion.

The dose in yellow and green is the smallest cefepime dosing regimen attaining PD targets of 60% fT>MIC of 8 mg/L and 60% fT>MICx4 of 32mg/L respectively.

**Table S5. PTA of various cefepime dosing regimens in KRT setting 4 (Late 9-hour PIKRT)**

| **Cefepime Dose** | **MIC**  **(mg/L)** | **PTA during 1-week of therapy** | | | | | | | |
| --- | --- | --- | --- | --- | --- | --- | --- | --- | --- |
|  |  | **Mean** | **Day 1** | **Day 2** | **Day 3** | **Day 4** | **Day 5** | **Day 6** | **Day 7** |
| 1g q24h post- PIKRT | 8 | 89.4% | 80.8% | 89.6% | 90.4% | 90.6% | 90.6% | 90.6% | 90.7% |
|  | 32 | 2.0% | 1.3% | 2.7% | 3.1% | 3.2% | 3.3% | 3.3% | 3.3% |
| 2g LD, then 1g q24h post-HD PIKRT | 8 | 91.9% | 98.9% | 92.4% | 91.1% | 91.0% | 91.0% | 91.0% | 90.9% |
|  | 32 | 3.9% | 28.6% | 5.7% | 4.0% | 3.7% | 3.6% | 3.6% | 3.6% |
| 1g q12h post- PIKRT | 8 | 99.5% | 95.0% | 99.5% | 99.5% | 99.5% | 99.5% | 99.5% | 99.5% |
|  | 32 | 17.2% | 4.0% | 13.6% | 18.8% | 21.3% | 22.5% | 22.5% | 23.1% |
| 2g q12h post- PIKRT | 8 | 100.0% | 100.0% | 100.0% | 100.0% | 100.0% | 100.0% | 100.0% | 100.0% |
|  | 32 | 83.6% | 48.7% | 80.9% | 85.1% | 85.9% | 86.1% | 86.1% | 86.2% |
| 1g q8h post- PIKRT | 8 | 100.0% | 100.0% | 100.0% | 100.0% | 100.0% | 100.0% | 100.0% | 100.0% |
|  | 32 | 58.4% | 13.4% | 49.6% | 60.6% | 63.7% | 64.8% | 64.8% | 65.5% |
| 2g q8h post- PIKRT | 8 | 100.0% | 100.0% | 100.0% | 100.0% | 100.0% | 100.0% | 100.0% | 100.0% |
|  | 32 | 98.5% | 83.6% | 98.4% | 98.8% | 98.8% | 98.8% | 98.8% | 98.8% |
| 2g LD, 2g q8h EI post- PIKRT | 8 | 100.0% | 100.0% | 100.0% | 100.0% | 100.0% | 100.0% | 100.0% | 100.0% |
|  | 32 | 99.5% | 80.0% | 99.3% | 99.6% | 99.6% | 99.6% | 99.6% | 99.6% |
| 3g LD, 2g q8h post-PIKRT | 8 | 100.0% | 100.0% | 100.0% | 100.0% | 100.0% | 100.0% | 100.0% | 100.0% |
|  | 32 | 99.0% | 94.8% | 98.9% | 99.0% | 99.1% | 99.1% | 99.1% | 99.1% |
| 1g q6h post-PIKRT | 8 | 100.0% | 100.0% | 100.0% | 100.0% | 100.0% | 100.0% | 100.0% | 100.0% |
|  | 32 | 85.2% | 42.7% | 81.9% | 87.1% | 88.1% | 88.4% | 88.4% | 88.3% |
| 1g LD, 1g q6h EI post-PIKRT | 8 | 100.0% | 99.8% | 100.0% | 100.0% | 100.0% | 100.0% | 100.0% | 100.0% |
|  | 32 | 82.6% | 13.7% | 79.8% | 85.4% | 86.3% | 86.6% | 86.6% | 86.7% |
| 2g LD, 1g q6h post-PIKRT | 8 | 100.0% | 100.0% | 100.0% | 100.0% | 100.0% | 100.0% | 100.0% | 100.0% |
|  | 32 | 87.9% | 77.4% | 85.9% | 87.5% | 88.0% | 88.2% | 88.2% | 88.1% |
| 2g LD, 1g q6h EI post-PIKRT | 8 | 100.0% | 100.0% | 100.0% | 100.0% | 100.0% | 100.0% | 100.0% | 100.0% |
|  | 32 | 87.2% | 74.7% | 84.7% | 86.8% | 87.3% | 87.4% | 87.4% | 87.5% |
| 3g LD, 1g q6h post-PIKRT | 8 | 100.0% | 100.0% | 100.0% | 100.0% | 100.0% | 100.0% | 100.0% | 100.0% |
|  | 32 | 89.5% | 92.5% | 91.7% | 88.8% | 88.5% | 88.5% | 88.5% | 88.5% |
| 3g LD, 1g q6h EI post-PIKRT | 8 | 100.0% | 100.0% | 100.0% | 100.0% | 100.0% | 100.0% | 100.0% | 100.0% |
|  | 32 | 88.7% | 91.1% | 87.7% | 87.6% | 87.6% | 87.6% | 87.6% | 87.6% |

MIC: minimum inhibitory concentration; PTA: probability of target attainment; 60%*f*T> MIC: 60% of a dosing interval that a free drug concentration exceeds the MIC.

EI: extended infusion over 4 hours. All other doses were simulated as a 30 min infusion.

The dose in yellow and green is the smallest cefepime dosing regimen attaining PD targets of 60% fT>MIC of 8 mg/L and 60% fT>MICx4 of 32mg/L respectively.

**Table S6. PTA of various cefepime dosing regimens in KRT setting 4 (Extended PIKRT)**

| **Cefepime Dose** | **MIC**  **(mg/L)** | **PTA during 1-week of therapy** | | | | | | | | |
| --- | --- | --- | --- | --- | --- | --- | --- | --- | --- | --- |
|  |  | **Mean** | **Day 1** | **Day 2** | **Day 3** | **Day 4** | **Day 5** | **Day 6** | | **Day 7** |
| 1g q24h | 8 | 23.4% | 7.0% | 22.3% | 26.4% | 27.4% | 27.7% | 27.7% | | 27.8% |
|  | 32 | 0.0% | 0.0% | 0.0% | 0.0% | 0.0% | 0.0% | 0.0% | | 0.0% |
| 1g q12h post-HD | 8 | 93.2% | 85.3% | 93.2% | 93.3% | 93.3% | 93.3% | 93.3% | | 93.3% |
|  | 32 | 0.4% | 0.0% | 0.2% | 0.6% | 0.8% | 0.9% | 0.9% | | 0.9% |
| 1g LD, 1g q12h EI | 8 | 98.8% | 88.3% | 98.8% | 98.8% | 98.8% | 98.8% | 98.8% | | 98.8% |
|  | 32 | 0.4% | 0.0% | 0.2% | 0.6% | 1.0% | 1.1% | 1.1% | | 1.2% |
| 2g LD, 1g q12h | 8 | 95.9% | 98.8% | 95.0% | 94.8% | 94.8% | 94.8% | 94.8% | 94.8% | |
|  | 32 | 0.9% | 1.4% | 0.7% | 0.9% | 1.0% | 1.1% | 1.1% | 1.2% | |
| 2g q12h | 8 | 99.5% | 99.5% | 99.5% | 99.5% | 99.5% | 99.5% | 99.5% | | 99.5% |
|  | 32 | 43.8% | 11.3% | 41.4% | 47.4% | 48.7% | 49.1% | 49.1% | | 49.2% |
| 1g q8h | 8 | 99.8% | 99.3% | 99.8% | 99.8% | 99.8% | 99.8% | 99.8% | | 99.8% |
|  | 32 | 18.1% | 1.0% | 14.9% | 20.8% | 23.0% | 23.7% | 23.7% | | 24.2% |
| 2g q8h | 8 | 100.0% | 100.0% | 100.0% | 100.0% | 100.0% | 100.0% | 100.0% | | 100.0% |
|  | 32 | 91.3% | 62.9% | 91.1% | 92.2% | 92.3% | 92.4% | 92.4% | | 92.4% |
| 2g LD, 2g q8h EI | 8 | 100.0% | 100.0% | 100.0% | 100.0% | 100.0% | 100.0% | 100.0% | | 100.0% |
|  | 32 | 95.8% | 61.6% | 95.4% | 96.5% | 96.6% | 96.6% | 96.6% | | 96.6% |
| 3g LD, 2g q8h EI | 8 | 100.0% | 100.0% | 100.0% | 100.0% | 100.0% | 100.0% | 100.0% | | 100.0% |
|  | 32 | 96.3% | 83.0% | 96.1% | 96.7% | 96.7% | 96.7% | 96.7% | | 96.8% |
| 3g-3g-2g on day 1, 2g q8h | 8 | 99.9% | 100.0% | 100.0% | 100.0% | 100.0% | 100.0% | 100.0% | | 100.0% |
|  | 32 | 91.7% | 93.3% | 91.8% | 91.7% | 91.7% | 91.7% | 91.7% | | 91.7% |
| 1g q6h | 8 | 100.0% | 99.9% | 100.0% | 100.0% | 100.0% | 100.0% | 100.0% | | 100.0% |
|  | 32 | 58.6% | 12.5% | 54.1% | 60.3% | 61.7% | 62.1% | 62.1% | | 62.1% |
| 1g LD, 1g q6h EI | 8 | 100.0% | 99.9% | 100.0% | 100.0% | 100.0% | 100.0% | 100.0% | | 100.0% |
|  | 32 | 69.9% | 12.4% | 62.6% | 71.6% | 72.6% | 72.9% | 72.9% | | 73.0% |

MIC: minimum inhibitory concentration; PTA: probability of target attainment; 60%*f*T> MIC: 60% of a dosing interval that a free drug concentration exceeds the MIC.

EI: extended infusion over 4 hours. All other doses were simulated as a 30 min infusion.

The dose in yellow and green is the smallest cefepime dosing regimen attaining PD targets of 60% fT>MIC of 8 mg/L and 60% fT>MICx4 of 32mg/L respectively.

## Ceftazidime simulation results

**Table S7. PTA of various ceftazidime dosing regimens in KRT setting 1 (Thrice-weekly HD)**

| **Ceftazidime Dose** | **MIC**  **(mg/L)** | **PTA during 1-week of therapy** | | | | | | | |
| --- | --- | --- | --- | --- | --- | --- | --- | --- | --- |
|  |  | **Mean** | **Day 1** | **Day 2** | **Day 3** | **Day 4** | **Day 5** | **Day 6** | **Day 7** |
| 0.5g q24h post-HD | 8 | 75.5% | 44.0% | 76.2% | 71.3% | 82.7% | 74.3% | 83.3% | 84.9% |
|  | 32 | 0.0% | 0.0% | 0.0% | 0.0% | 0.0% | 0.0% | 0.0% | 0.0% |
| 1g q24h post-HD | 8 | 98.9% | 96.3% | 99.0% | 98.9% | 99.0% | 98.9% | 99.0% | 99.0% |
|  | 32 | 4.1% | 0.3% | 6.9% | 1.4% | 11.5% | 2.0% | 13.0% | 19.3% |
| 2g q24h post-HD | 8 | 100.0% | 100.0% | 100.0% | 100.0% | 100.0% | 100.0% | 100.0% | 100.0% |
|  | 32 | 76.4% | 45.5% | 76.7% | 71.8% | 82.9% | 75.3% | 83.7% | 85.1% |
| 2g LD, 1g q12h post-HD | 8 | 100.0% | 100.0% | 100.0% | 100.0% | 100.0% | 100.0% | 100.0% | 100.0% |
|  | 32 | 89.2% | 83.0% | 90.4% | 78.4% | 91.9% | 79.9% | 92.4% | 93.5% |
| 2g q12h post-HD | 8 | 100.0% | 100.0% | 100.0% | 100.0% | 100.0% | 100.0% | 100.0% | 100.0% |
|  | 32 | 100.0% | 86.9% | 99.9% | 100.0% | 100.0% | 100.0% | 100.0% | 100.0% |
| 1g q8h post-HD | 8 | 100.0% | 100.0% | 100.0% | 100.0% | 100.0% | 100.0% | 100.0% | 100.0% |
|  | 32 | 99.9% | 71.9% | 98.1% | 99.5% | 99.9% | 99.8% | 100.0% | 99.9% |
| 2g LD, 1g q8h post-HD | 8 | 100.0% | 100.0% | 100.0% | 100.0% | 100.0% | 100.0% | 100.0% | 100.0% |
|  | 32 | 99.9% | 92.6% | 99.5% | 99.7% | 100.0% | 99.8% | 100.0% | 100.0% |

MIC: minimum inhibitory concentration; PTA: probability of target attainment; 60%*f*T> MIC: 60% of a dosing interval that a free drug concentration exceeds the MIC. All doses were simulated as a 30 min infusion.

The dose in yellow and green is the smallest ceftazidime dosing regimen attaining PD targets of 60% fT>MIC of 8 mg/L and 60% fT>MICx4 of 32mg/L respectively.

**Table S8. PTA of various ceftazidime dosing regimens in KRT setting 2 (Daily HD)**

| **Ceftazidime Dose** | **MIC**  **(mg/L)** | **PTA during 1-week of therapy** | | | | | | | |
| --- | --- | --- | --- | --- | --- | --- | --- | --- | --- |
|  |  | **Mean** | **Day 1** | **Day 2** | **Day 3** | **Day 4** | **Day 5** | **Day 6** | **Day 7** |
| 0.5g q24h post-HD | 8 | 65.9% | 43.4% | 63.6% | 68.9% | 70.6% | 71.2% | 71.5% | 71.6% |
|  | 32 | 0.0% | 0.0% | 0.0% | 0.0% | 0.0% | 0.0% | 0.0% | 0.0% |
| 1g q24h post-HD | 8 | 98.8% | 96.4% | 98.7% | 98.8% | 98.8% | 98.8% | 98.8% | 98.8% |
|  | 32 | 0.9% | 0.3% | 1.0% | 1.3% | 1.3% | 1.3% | 1.3% | 1.3% |
| 2g q24h post-HD | 8 | 99.9% | 99.9% | 99.9% | 99.9% | 99.9% | 99.9% | 99.9% | 99.9% |
|  | 32 | 66.9% | 43.8% | 63.8% | 69.4% | 71.3% | 71.8% | 72.1% | 72.3% |
| 2g LD, 1g q12h post-HD | 8 | 100.0% | 100.0% | 100.0% | 100.0% | 100.0% | 100.0% | 100.0% | 100.0% |
|  | 32 | 70.5% | 81.9% | 66.7% | 65.4% | 66.0% | 66.5% | 66.9% | 67.0% |
| 2g q12h post-HD | 8 | 100.0% | 100.0% | 100.0% | 100.0% | 100.0% | 100.0% | 100.0% | 100.0% |
|  | 32 | 100.0% | 84.2% | 99.6% | 100.0% | 100.0% | 100.0% | 100.0% | 100.0% |
| 1g q8h | 8 | 100.0% | 100.0% | 100.0% | 100.0% | 100.0% | 100.0% | 100.0% | 100.0% |
|  | 32 | 97.0% | 56.1% | 92.6% | 97.0% | 98.2% | 98.6% | 98.5% | 99.0% |
| 2g LD, 1g q8h | 8 | 100.0% | 100.0% | 100.0% | 100.0% | 100.0% | 100.0% | 100.0% | 100.0% |
|  | 32 | 98.7% | 90.8% | 96.7% | 98.1% | 98.6% | 98.8% | 98.7% | 98.9% |

MIC: minimum inhibitory concentration; PTA: probability of target attainment; 60%*f*T> MIC: 60% of a dosing interval that a free drug concentration exceeds the MIC. All doses were simulated as a 30 min infusion.

The dose in yellow and green is the smallest ceftazidime dosing regimen attaining PD targets of 60% fT>MIC of 8 mg/L and 60% fT>MICx4 of 32mg/L respectively.

**Table S9. PTA of various ceftazidime dosing regimens in KRT setting 3 (Sequential therapy)**

| **Ceftazidime Dose** | **MIC**  **(mg/L)** | **PTA during 1-week of therapy** | | | | | | | |
| --- | --- | --- | --- | --- | --- | --- | --- | --- | --- |
|  |  | **Mean** | **Day 1** | **Day 2** | **Day 3** | **Day 4** | **Day 5** | **Day 6** | **Day 7** |
| 0.5g q24h post-HD | 8 | 41.1% | 24.1% | 39.9% | 44.9% | 47.0% | 47.9% | 48.3% | 48.4% |
|  | 32 | 0.0% | 0.0% | 0.0% | 0.0% | 0.0% | 0.0% | 0.0% | 0.0% |
| 1g q24h post-HD | 8 | 96.5% | 93.0% | 96.4% | 96.5% | 96.5% | 96.5% | 96.5% | 96.5% |
|  | 32 | 0.0% | 0.0% | 0.0% | 0.0% | 0.0% | 0.0% | 0.0% | 0.0% |
| 2g q24h post-HD | 8 | 99.8% | 99.8% | 99.8% | 99.8% | 99.8% | 99.8% | 99.8% | 99.8% |
|  | 32 | 44.3% | 24.8% | 42.8% | 48.2% | 49.8% | 50.4% | 50.7% | 51.0% |
| 2g LD, 1g q12h post-HD | 8 | 100.0% | 100.0% | 100.0% | 100.0% | 100.0% | 100.0% | 100.0% | 100.0% |
|  | 32 | 51.5% | 75.1% | 47.8% | 45.5% | 46.2% | 46.6% | 46.7% | 46.8% |
| 2g q12h post-HD | 8 | 100.0% | 100.0% | 100.0% | 100.0% | 100.0% | 100.0% | 100.0% | 100.0% |
|  | 32 | 99.9% | 82.4% | 99.2% | 99.9% | 99.9% | 99.9% | 99.9% | 99.9% |
| 1g q8h | 8 | 100.0% | 100.0% | 100.0% | 100.0% | 100.0% | 100.0% | 100.0% | 100.0% |
|  | 32 | 93.7% | 49.0% | 88.9% | 94.5% | 95.8% | 96.0% | 96.2% | 96.8% |
| 2g LD, 1g q8h | 8 | 100.0% | 100.0% | 100.0% | 100.0% | 100.0% | 100.0% | 100.0% | 100.0% |
|  | 32 | 95.9% | 88.3% | 92.6% | 94.8% | 95.5% | 95.6% | 95.8% | 96.5% |

MIC: minimum inhibitory concentration; PTA: probability of target attainment; 60%*f*T> MIC: 60% of a dosing interval that a free drug concentration exceeds the MIC. All doses were simulated as a 30 min infusion.

The dose in yellow and green is the smallest ceftazidime dosing regimen attaining PD targets of 60% fT>MIC of 8 mg/L and 60% fT>MICx4 of 32mg/L respectively.

**Table S10. PTA of various ceftazidime dosing regimens in KRT setting 4 (Early 9-hour PIKRT)**

| **Ceftazidime Dose** | **MIC**  **(mg/L)** | **PTA during 1-week of therapy** | | | | | | | |
| --- | --- | --- | --- | --- | --- | --- | --- | --- | --- |
|  |  | **Mean** | **Day 1** | **Day 2** | **Day 3** | **Day 4** | **Day 5** | **Day 6** | **Day 7** |
| 1g q24h | 8 | 25.4% | 5.9% | 23.1% | 27.2% | 27.9% | 28.2% | 28.3% | 28.3% |
|  | 32 | 0.0% | 0.0% | 0.0% | 0.0% | 0.0% | 0.0% | 0.0% | 0.0% |
| 2g q24h | 8 | 65.8% | 64.1% | 66.3% | 66.4% | 66.4% | 66.4% | 66.4% | 66.4% |
|  | 32 | 0.1% | 0.0% | 0.0% | 0.1% | 0.3% | 0.3% | 0.5% | 0.6% |
| 1g q12h | 8 | 100.0% | 98.4% | 99.8% | 99.8% | 99.8% | 99.8% | 99.8% | 99.8% |
|  | 32 | 13.9% | 1.7% | 14.2% | 18.4% | 20.0% | 20.5% | 20.8% | 20.9% |
| 2g q12h | 8 | 100.0% | 100.0% | 100.0% | 100.0% | 100.0% | 100.0% | 100.0% | 100.0% |
|  | 32 | 96.7% | 72.2% | 96.4% | 97.2% | 97.3% | 97.3% | 97.3% | 97.3% |
| 1g q8h | 8 | 100.0% | 100.0% | 100.0% | 100.0% | 100.0% | 100.0% | 100.0% | 100.0% |
|  | 32 | 83.9% | 29.2% | 78.6% | 86.4% | 88.1% | 88.3% | 90.7% | 90.5% |
| 2g LD, 1g q8h | 8 | 100.0% | 100.0% | 100.0% | 100.0% | 100.0% | 100.0% | 100.0% | 100.0% |
|  | 32 | 86.7% | 67.6% | 83.7% | 86.7% | 87.6% | 88.0% | 90.8% | 90.4% |
| 3g LD, 1g q8h | 8 | 100.0% | 100.0% | 100.0% | 100.0% | 100.0% | 100.0% | 100.0% | 100.0% |
|  | 32 | 90.3% | 90.4% | 88.0% | 88.7% | 88.9% | 88.9% | 91.2% | 91.0% |
| 2g q8h | 8 | 100.0% | 100.0% | 100.0% | 100.0% | 100.0% | 100.0% | 100.0% | 100.0% |
|  | 32 | 100.0% | 95.2% | 100.0% | 100.0% | 100.0% | 100.0% | 100.0% | 100.0% |
| 1g q6h | 8 | 100.0% | 100.0% | 100.0% | 100.0% | 100.0% | 100.0% | 100.0% | 100.0% |
|  | 32 | 99.7% | 46.5% | 98.6% | 99.9% | 100.0% | 100.0% | 99.9% | 99.9% |
| 2g LD, 1g q6h | 8 | 100.0% | 100.0% | 100.0% | 100.0% | 100.0% | 100.0% | 100.0% | 100.0% |
|  | 32 | 99.6% | 85.2% | 99.2% | 99.7% | 99.7% | 99.8% | 99.7% | 99.8% |

MIC: minimum inhibitory concentration; PTA: probability of target attainment; 60%*f*T> MIC: 60% of a dosing interval that a free drug concentration exceeds the MIC. All doses were simulated as a 30 min infusion.

The dose in yellow and green is the smallest ceftazidime dosing regimen attaining PD targets of 60% fT>MIC of 8 mg/L and 60% fT>MICx4 of 32mg/L respectively.

**Table S11. PTA of various ceftazidime dosing regimens in KRT setting 5 (Late 9-hour PIKRT)**

| **Ceftazidime Dose** | **MIC**  **(mg/L)** | **PTA during 1-week of therapy** | | | | | | | |
| --- | --- | --- | --- | --- | --- | --- | --- | --- | --- |
|  |  | **Mean** | **Day 1** | **Day 2** | **Day 3** | **Day 4** | **Day 5** | **Day 6** | **Day 7** |
| 1g q24h post-PIKRT | 8 | 98.7% | 96.1% | 98.7% | 98.7% | 98.7% | 98.7% | 98.7% | 98.7% |
|  | 32 | 0.6% | 0.3% | 0.6% | 0.8% | 0.8% | 0.8% | 0.8% | 0.8% |
| 2g q24h post-PIKRT | 8 | 99.9% | 99.9% | 99.9% | 99.9% | 99.9% | 99.9% | 99.9% | 99.9% |
|  | 32 | 57.5% | 42.8% | 60.9% | 65.3% | 66.8% | 67.5% | 67.7% | 67.9% |
| 1g q12h post-PIKRT | 8 | 100.0% | 99.0% | 100.0% | 100.0% | 100.0% | 100.0% | 100.0% | 100.0% |
|  | 32 | 23.9% | 9.6% | 24.1% | 29.7% | 32.2% | 33.1% | 33.3% | 33.6% |
| 2g q12h post-PIKRT | 8 | 100.0% | 100.0% | 100.0% | 100.0% | 100.0% | 100.0% | 100.0% | 100.0% |
|  | 32 | 98.9% | 80.3% | 97.9% | 99.1% | 99.1% | 99.1% | 99.1% | 99.1% |
| 1g q8h post-PIKRT | 8 | 100.0% | 100.0% | 100.0% | 100.0% | 100.0% | 100.0% | 100.0% | 100.0% |
|  | 32 | 87.8% | 39.1% | 83.4% | 90.2% | 92.1% | 92.4% | 92.5% | 92.3% |
| 2g LD, 1g q8h post-PIKRT | 8 | 100.0% | 100.0% | 100.0% | 100.0% | 100.0% | 100.0% | 100.0% | 100.0% |
|  | 32 | 93.4% | 88.1% | 90.8% | 92.3% | 92.7% | 92.8% | 92.9% | 92.7% |
| 3g LD, 1g q8h post-PIKRT | 8 | 100.0% | 100.0% | 100.0% | 100.0% | 100.0% | 100.0% | 100.0% | 100.0% |
|  | 32 |  | 96.6% | 94.3% | 93.1% | 93.2% | 93.2% | 93.3% | 93.0% |
| 2g q8h post-PIKRT | 8 | 100.0% | 100.0% | 100.0% | 100.0% | 100.0% | 100.0% | 100.0% | 100.0% |
|  | 32 | 100.0% | 97.6% | 100.0% | 100.0% | 100.0% | 100.0% | 100.0% | 100.0% |
| 1g q6h post-PIKRT | 8 | 100.0% | 100.0% | 100.0% | 100.0% | 100.0% | 100.0% | 100.0% | 100.0% |
|  | 32 | 99.9% | 78.8% | 99.0% | 99.9% | 100.0% | 100.0% | 100.0% | 100.0% |
| 2g LD, 1g q6h post-PIKRT | 8 | 100.0% | 100.0% | 100.0% | 100.0% | 100.0% | 100.0% | 100.0% | 100.0% |
|  | 32 | 99.9% | 94.3% | 99.5% | 99.9% | 99.9% | 99.9% | 99.9% | 99.9% |

MIC: minimum inhibitory concentration; PTA: probability of target attainment; 60%*f*T> MIC: 60% of a dosing interval that a free drug concentration exceeds the MIC. All doses were simulated as a 30 min infusion.

The dose in yellow and green is the smallest ceftazidime dosing regimen attaining PD targets of 60% fT>MIC of 8 mg/L and 60% fT>MICx4 of 32mg/L respectively.

**Table S12. PTA of various ceftazidime dosing regimens in KRT setting 6 (Extended PIKRT)**

| **Ceftazidime Dose** | **MIC**  **(mg/L)** | **PTA during 1-week of therapy** | | | | | | | |
| --- | --- | --- | --- | --- | --- | --- | --- | --- | --- |
|  |  | **Mean** | **Day 1** | **Day 2** | **Day 3** | **Day 4** | **Day 5** | **Day 6** | **Day 7** |
| 1g q24h | 8 | 15.7% | 5.1% | 16.1% | 18.7% | 19.4% | 19.5% | 19.6% | 19.6% |
|  | 32 | 0.0% | 0.0% | 0.0% | 0.0% | 0.0% | 0.0% | 0.0% | 0.0% |
| 2g q24h | 8 | 67.5% | 66.3% | 67.5% | 67.6% | 67.6% | 67.6% | 67.6% | 67.6% |
|  | 32 | 0.0% | 0.0% | 0.0% | 0.0% | 0.0% | 0.0% | 0.0% | 0.0% |
| 1g q12h | 8 | 95.4% | 94.2% | 95.4% | 95.4% | 95.4% | 95.4% | 95.4% | 95.4% |
|  | 32 | 0.0% | 0.0% | 0.0% | 0.1% | 0.1% | 0.1% | 0.1% | 0.1% |
| 2g q12h | 8 | 99.6% | 99.6% | 99.6% | 99.6% | 99.6% | 99.6% | 99.6% | 99.6% |
|  | 32 | 47.1% | 16.4% | 46.4% | 49.6% | 50.0% | 50.1% | 50.2% | 50.2% |
| 1g q8h | 8 | 100.0% | 100.0% | 100.0% | 100.0% | 100.0% | 100.0% | 100.0% | 100.0% |
|  | 32 | 17.4% | 1.2% | 16.9% | 21.9% | 23.2% | 23.5% | 23.9% | 25.6% |
| 2g LD, 1g q8h | 8 | 100.0% | 100.0% | 100.0% | 100.0% | 100.0% | 100.0% | 100.0% | 100.0% |
|  | 32 | 21.5% | 30.8% | 20.5% | 22.0% | 22.6% | 22.8% | 23.1% | 25.2% |
| 2g q8h | 8 | 100.0% | 100.0% | 100.0% | 100.0% | 100.0% | 100.0% | 100.0% | 100.0% |
|  | 32 | 97.4% | 87.2% | 97.3% | 97.4% | 97.4% | 97.4% | 97.4% | 97.4% |
| 1g q6h | 8 | 100.0% | 100.0% | 100.0% | 100.0% | 100.0% | 100.0% | 100.0% | 100.0% |
|  | 32 | 72.9% | 26.5% | 71.0% | 74.4% | 74.8% | 74.9% | 74.9% | 0.0% |

MIC: minimum inhibitory concentration; PTA: probability of target attainment; 60%*f*T> MIC: 60% of a dosing interval that a free drug concentration exceeds the MIC. All doses were simulated as a 30 min infusion.

The dose in yellow and green is the smallest ceftazidime dosing regimen attaining PD targets of 60% fT>MIC of 8 mg/L and 60% fT>MICx4 of 32mg/L respectively.

1. **Imipenem Simulation Results**

**Table S13. PTA of various imipenem dosing regimens in KRT setting 1 (Thrice-weekly HD)**

| **Imipenem Dose** | **MIC**  **(mg/L)** | **PTA during 1-week of therapy** | | | | | | | |
| --- | --- | --- | --- | --- | --- | --- | --- | --- | --- |
|  |  | **Mean** | **Day 1** | **Day 2** | **Day 3** | **Day 4** | **Day 5** | **Day 6** | **Day 7** |
| 500 mg LD, 250 mg q12h post-HD | 2 | 79.3% | 90.3% | 79.1% | 77.0% | 79.1% | 77.0% | 79.1% | 79.2% |
|  | 8 | 0.2% | 1.8% | 0.2% | 0.0% | 0.2% | 0.0% | 0.2% | 0.3% |
| 500 mg q12h post-HD | 2 | 96.8% | 96.8% | 96.8% | 96.8% | 96.8% | 96.8% | 96.8% | 96.8% |
|  | 8 | 18.5% | 9.2% | 19.3% | 14.9% | 21.4% | 15.1% | 21.4% | 22.1% |
| 1g LD, 500 mg q8h post-HD | 2 | 99.9% | 99.9% | 99.9% | 99.9% | 99.9% | 99.9% | 99.9% | 99.9% |
|  | 8 | 67.4% | 81.9% | 67.8% | 63.9% | 67.5% | 63.9% | 67.3% | 67.9% |
| 750 mg q8h post-HD | 2 | 99.9% | 99.9% | 99.9% | 99.9% | 99.9% | 99.9% | 99.9% | 99.9% |
|  | 8 | 92.2% | 89.4% | 92.4% | 91.9% | 92.5% | 91.9% | 92.5% | 92.5% |
| 1g LD, 750 mg q8h post-HD | 2 | 99.9% | 99.9% | 99.9% | 99.9% | 99.9% | 99.9% | 99.9% | 99.9% |
|  | 8 | 92.5% | 92.4% | 92.6% | 92.0% | 92.6% | 92.0% | 92.6% | 92.6% |
| 1g LD, 500 mg q6h post-HD | 2 | 100.0% | 100.0% | 99.9% | 100.0% | 99.9% | 100.0% | 99.9% | 99.9% |
|  | 8 | 90.0% | 93.9% | 84.0% | 85.7% | 83.0% | 83.6% | 83.0% | 84.0% |

MIC: minimum inhibitory concentration; PTA: probability of target attainment; 40%*f*T> MIC: 40% of a dosing interval that a free drug concentration exceeds the MIC. All doses were simulated as a 30 min infusion.

The dose in yellow and green is the smallest imipenem dosing regimen attaining PD targets of 40% fT>MIC of 2 mg/L and 40% fT>MICx4 of 8mg/L respectively.

**Table S14. PTA of various imipenem d osing regimens in KRT setting 2 (Daily HD)**

| **Imipenem Dose** | **MIC**  **(mg/L)** | **PTA during 1-week of therapy** | | | | | | | |
| --- | --- | --- | --- | --- | --- | --- | --- | --- | --- |
|  |  | **Mean** | **Day 1** | **Day 2** | **Day 3** | **Day 4** | **Day 5** | **Day 6** | **Day 7** |
| 500 mg q12h post-HD | 2 | 96.7% | 96.7% | 96.7% | 96.7% | 96.7% | 96.7% | 96.7% | 96.7% |
|  | 8 | 14.5% | 9.8% | 14.5% | 15.0% | 15.2% | 15.2% | 15.2% | 15.2% |
| 1g LD, 500 mg q8h post-HD | 2 | 99.8% | 99.9% | 99.8% | 99.8% | 99.8% | 99.8% | 99.8% | 99.8% |
|  | 8 | 66.3% | 82.7% | 64.6% | 64.5% | 64.9% | 65.2% | 64.9% | 65.2% |
| 750 mg q8h post-HD | 2 | 100.0% | 99.9% | 99.9% | 99.9% | 99.9% | 99.9% | 99.9% | 99.9% |
|  | 8 | 91.4% | 88.7% | 91.4% | 91.6% | 91.6% | 91.7% | 91.6% | 91.7% |
| 1g LD, 750 mg q8h post-HD | 2 | 99.9% | 99.9% | 99.9% | 99.9% | 99.9% | 99.9% | 99.9% | 99.9% |
|  | 8 | 91.9% | 92.2% | 91.7% | 91.7% | 91.7% | 91.8% | 91.7% | 91.8% |
| 1g LD, 500 mg q6h post-HD | 2 | 100.0% | 100.0% | 99.9% | 99.9% | 99.9% | 99.9% | 99.9% | 99.9% |
|  | 8 | 85.0% | 93.0% | 81.6% | 81.5% | 81.5% | 81.5% | 81.5% | 81.5% |

MIC: minimum inhibitory concentration; PTA: probability of target attainment; 40%*f*T> MIC: 40% of a dosing interval that a free drug concentration exceeds the MIC. All doses were simulated as a 30 min infusion.

The dose in yellow and green is the smallest imipenem dosing regimen attaining PD targets of 40% fT>MIC of 2 mg/L and 40% fT>MICx4 of 8mg/L respectively.

**Table S15. PTA of various imipenem dosing regimens in KRT setting 3 (Sequential therapy)**

| **Imipenem Dose** | **MIC**  **(mg/L)** | **PTA during 1-week of therapy** | | | | | | | |
| --- | --- | --- | --- | --- | --- | --- | --- | --- | --- |
|  |  | **Mean** | **Day 1** | **Day 2** | **Day 3** | **Day 4** | **Day 5** | **Day 6** | **Day 7** |
| 500 mg q12h post-HD | 2 | 96.5% | 96.4% | 96.5% | 96.5% | 96.5% | 96.5% | 96.5% | 96.5% |
|  | 8 | 11.2% | 7.1% | 11.2% | 11.6% | 11.6% | 11.6% | 11.6% | 11.6% |
| 1g LD, 500 mg q8h post-HD | 2 | 99.7% | 99.7% | 99.7% | 99.7% | 99.7% | 99.7% | 99.7% | 99.7% |
|  | 8 | 61.3% | 79.4% | 59.3% | 59.0% | 59.4% | 59.4% | 59.4% | 59.7% |
| 750 mg q8h post-HD | 2 | 99.9% | 99.9% | 99.9% | 99.9% | 99.9% | 99.9% | 99.9% | 99.9% |
|  | 8 | 90.2% | 86.8% | 90.2% | 90.3% | 90.3% | 90.3% | 90.3% | 90.5% |
| 1g LD, 750 mg q8h post-HD | 2 | 99.9% | 99.9% | 99.9% | 99.9% | 99.9% | 99.9% | 99.9% | 99.9% |
|  | 8 | 90.5% | 90.7% | 90.4% | 90.4% | 90.4% | 90.4% | 90.4% | 90.4% |
| 1g LD, 500 mg q6h post-HD | 2 | 99.9% | 99.9% | 99.9% | 99.9% | 99.9% | 99.9% | 99.9% | 99.9% |
|  | 8 | 82.3% | 91.3% | 77.9% | 77.8% | 77.8% | 77.8% | 77.8% | 77.8% |

MIC: minimum inhibitory concentration; PTA: probability of target attainment; 40%*f*T> MIC: 40% of a dosing interval that a free drug concentration exceeds the MIC. All doses were simulated as a 30 min infusion.

The dose in yellow and green is the smallest imipenem dosing regimen attaining PD targets of 40% fT>MIC of 2 mg/L and 40% fT>MICx4 of 8mg/L respectively.

**Table S16. PTA of various imipenem dosing regimens in KRT setting 4 (Early 9-hour PIKRT)**

| **Imipenem Dose** | **MIC**  **(mg/L)** | **PTA during 1-week of therapy** | | | | | | | |
| --- | --- | --- | --- | --- | --- | --- | --- | --- | --- |
|  |  | **Mean** | **Day 1** | **Day 2** | **Day 3** | **Day 4** | **Day 5** | **Day 6** | **Day 7** |
| 500 mg q12h | 2 | 89.8% | 89.6% | 89.8% | 89.8% | 89.8% | 89.8% | 89.8% | 89.8% |
|  | 8 | 3.5% | 1.2% | 3.4% | 3.7% | 3.8% | 3.8% | 3.8% | 3.8% |
| 1g LD, 500 mg q8h | 2 | 99.5% | 99.6% | 99.5% | 99.5% | 99.5% | 99.5% | 99.5% | 99.5% |
|  | 8 | 46.9% | 55.1% | 43.6% | 43.5% | 43.5% | 43.5% | 43.5% | 43.9% |
| 750 mg q8h | 2 | 99.7% | 99.7% | 99.7% | 99.7% | 99.7% | 99.7% | 99.7% | 99.7% |
|  | 8 | 81.3% | 76.8% | 81.5% | 81.7% | 81.7% | 81.7% | 81.7% | 81.7% |
| 1g q8h | 2 | 99.8% | 99.8% | 99.8% | 99.8% | 99.8% | 99.8% | 99.8% | 99.8% |
|  | 8 | 94.1% | 93.3% | 94.2% | 94.2% | 94.2% | 94.2% | 94.2% | 94.2% |
| 1g LD, 500 mg q6h | 2 | 100.0% | 100.0% | 100.0% | 100.0% | 100.0% | 100.0% | 100.0% | 100.0% |
|  | 8 | 73.8% | 78.1% | 71.8% | 71.9% | 71.9% | 71.9% | 71.9% | 71.9% |
| 750 mg q6h | 2 | 100.0% | 100.0% | 100.0% | 100.0% | 100.0% | 100.0% | 100.0% | 100.0% |
|  | 8 | 96.1% | 94.4% | 96.2% | 96.2% | 96.2% | 96.2% | 96.2% | 96.2% |

MIC: minimum inhibitory concentration; PTA: probability of target attainment; 40%*f*T> MIC: 40% of a dosing interval that a free drug concentration exceeds the MIC. All doses were simulated as a 30 min infusion.

The dose in yellow and green is the smallest imipenem dosing regimen attaining PD targets of 40% fT>MIC of 2 mg/L and 40% fT>MICx4 of 8mg/L respectively.

**Table S17. PTA of various imipenem dosing regimens in KRT setting 5 (Late 9-hour PIKRT)**

| **Imipenem Dose** | **MIC**  **(mg/L)** | **PTA during 1-week of therapy** | | | | | | | |
| --- | --- | --- | --- | --- | --- | --- | --- | --- | --- |
|  |  | **Mean** | **Day 1** | **Day 2** | **Day 3** | **Day 4** | **Day 5** | **Day 6** | **Day 7** |
| 500 mg q12h post-PIKRT | 2 | 95.1% | 95.1% | 95.1% | 95.1% | 95.1% | 95.1% | 95.1% | 95.1% |
|  | 8 | 6.8% | 4.2% | 6.8% | 7.1% | 7.2% | 7.2% | 7.1% | 7.2% |
| 1g LD, 500 mg q8h post-PIKRT | 2 | 99.9% | 99.9% | 99.6% | 99.6% | 99.6% | 99.6% | 99.6% | 99.6% |
|  | 8 | 54.8% | 74.4% | 49.7% | 49.2% | 49.2% | 49.2% | 49.3% | 49.8% |
| 750 mg q8h post-PIKRT | 2 | 99.8% | 99.8% | 99.8% | 99.8% | 99.8% | 99.8% | 99.8% | 99.8% |
|  | 8 | 85.6% | 80.0% | 85.2% | 85.2% | 85.2% | 85.2% | 85.2% | 85.3% |
| 1g q8h post-PIKRT | 2 | 99.9% | 99.9% | 99.9% | 99.9% | 99.9% | 99.9% | 99.9% | 99.9% |
|  | 8 | 95.1% | 94.5% | 95.0% | 95.1% | 95.1% | 95.1% | 95.1% | 95.1% |
| 1g LD, 500 mg q6h post-PIKRT | 2 | 99.9% | 100.0% | 99.9% | 99.9% | 99.9% | 99.9% | 99.9% | 99.9% |
|  | 8 | 79.1% | 90.2% | 78.6% | 76.7% | 76.6% | 76.6% | 76.6% | 76.8% |
| 750 mg q6h post-PIKRT | 2 | 99.9% | 100.0% | 99.9% | 99.9% | 99.9% | 99.9% | 99.9% | 99.9% |
|  | 8 | 97.0% | 96.5% | 97.2% | 97.1% | 97.1% | 97.1% | 97.1% | 97.1% |

MIC: minimum inhibitory concentration; PTA: probability of target attainment; 40%*f*T> MIC: 40% of a dosing interval that a free drug concentration exceeds the MIC. All doses were simulated as a 30 min infusion.

The dose in yellow and green is the smallest imipenem dosing regimen attaining PD targets of 40% fT>MIC of 2 mg/L and 40% fT>MICx4 of 8mg/L respectively.

**Table S18. PTA of various imipenem dosing regimens in KRT setting 6 (Extended PIKRT)**

| **Imipenem Dose** | **MIC**  **(mg/L)** | **PTA during 1-week of therapy** | | | | | | | |
| --- | --- | --- | --- | --- | --- | --- | --- | --- | --- |
|  |  | **Mean** | **Day 1** | **Day 2** | **Day 3** | **Day 4** | **Day 5** | **Day 6** | **Day 7** |
| 500 mg q12h | 2 | 87.5% | 87.2% | 87.5% | 87.5% | 87.5% | 87.5% | 87.5% | 87.5% |
|  | 8 | 1.0% | 0.2% | 1.1% | 1.3% | 1.3% | 1.3% | 1.3% | 1.3% |
| 500 mg q8h | 2 | 98.5% | 98.5% | 98.5% | 98.5% | 98.5% | 98.5% | 98.5% | 98.5% |
|  | 8 | 22.8% | 13.7% | 22.8% | 22.9% | 23.0% | 23.0% | 23.0% | 23.1% |
| 750 mg q8h | 2 | 99.5% | 99.5% | 99.5% | 99.5% | 99.5% | 99.5% | 99.5% | 99.5% |
|  | 8 | 67.1% | 60.9% | 67.0% | 67.1% | 67.1% | 67.1% | 67.1% | 67.1% |
| 1g q8h | 2 | 99.6% | 99.6% | 99.6% | 99.6% | 99.6% | 99.6% | 99.6% | 99.6% |
|  | 8 | 87.4% | 86.0% | 87.4% | 87.4% | 87.4% | 87.4% | 87.4% | 87.4% |
| 1g LD, 500 mg q6h | 2 | 100.0% | 100.0% | 100.0% | 100.0% | 100.0% | 100.0% | 100.0% | 100.0% |
|  | 8 | 66.9% | 74.6% | 66.9% | 67.0% | 67.0% | 67.0% | 67.0% | 67.0% |
| 750 mg q6h | 2 | 99.9% | 99.9% | 99.9% | 99.9% | 99.9% | 99.9% | 99.9% | 99.9% |
|  | 8 | 95.3% | 91.5% | 95.5% | 95.5% | 95.5% | 95.5% | 95.5% | 95.5% |

MIC: minimum inhibitory concentration; PTA: probability of target attainment; 40%*f*T> MIC: 40% of a dosing interval that a free drug concentration exceeds the MIC. All doses were simulated as a 30 min infusion.

The dose in yellow and green is the smallest imipenem dosing regimen attaining PD targets of 40% fT>MIC of 2 mg/L and 40% fT>MICx4 of 8mg/L respectively.

1. **Meropenem Simulation Results**

**Table S19. PTA of various meropenem dosing regimens in KRT setting 1 (Thrice-weekly HD)**

| **Meropenem Dose** | **MIC**  **(mg/L)** | **PTA during 1-week of therapy** | | | | | | | |
| --- | --- | --- | --- | --- | --- | --- | --- | --- | --- |
|  |  | **Mean** | **Day 1** | **Day 2** | **Day 3** | **Day 4** | **Day 5** | **Day 6** | **Day 7** |
| 0.5 g q24h post-HD | 2 | 97.3% | 97.2% | 97.3% | 97.3% | 97.3% | 97.3% | 96.7% | 97.3% |
|  | 8 | 56.9% | 36.1% | 56.1% | 53.2% | 59.9% | 54.4% | 58.5% | 61.7% |
| 1g q24h post-HD | 2 | 99.1% | 99.1% | 99.1% | 99.1% | 99.1% | 99.1% | 98.8% | 99.1% |
|  | 8 | 88.9% | 84.3% | 88.9% | 88.5% | 89.2% | 88.6% | 87.8% | 89.4% |
| 0.5g q12h post-HD | 2 | 99.9% | 99.9% | 99.9% | 99.9% | 99.9% | 99.9% | 99.8% | 99.9% |
|  | 8 | 93.0% | 83.3% | 92.9% | 91.7% | 93.8% | 91.8% | 92.0% | 93.9% |
| 1g LD, 0.5g q12h post-HD | 2 | 99.9% | 100.0% | 99.9% | 99.9% | 99.9% | 99.9% | 99.9% | 99.9% |
|  | 8 | 94.2% | 97.6% | 94.1% | 92.1% | 94.2% | 92.1% | 92.3% | 94.3% |
| 1g q12h post-HD | 2 | 100.0% | 100.0% | 100.0% | 100.0% | 100.0% | 100.0% | 100.0% | 100.0% |
|  | 8 | 99.7% | 99.6% | 99.7% | 99.7% | 99.7% | 99.7% | 99.4% | 99.7% |

MIC: minimum inhibitory concentration; PTA: probability of target attainment; 40%*f*T> MIC: 40% of a dosing interval that a free drug concentration exceeds the MIC. All doses were simulated as a 30 min infusion.

The dose in yellow and green is the smallest meropenem dosing regimen attaining PD targets of 40% fT>MIC of 2 mg/L and 40% fT>MICx4 of 8mg/L respectively.

**Table S20. PTA of various meropenem dosing regimens in KRT setting 2 (Daily HD)**

| **Meropenem Dose** | **MIC**  **(mg/L)** | **PTA during 1-week of therapy** | | | | | | | |
| --- | --- | --- | --- | --- | --- | --- | --- | --- | --- |
|  |  | **Mean** | **Day 1** | **Day 2** | **Mean** | **Day 4** | **Day 5** | **Mean** | **Day 7** |
| 0.5 mg q24h post-HD | 2 | 97.0% | 97.0% | 97.0% | 97.0% | 97.0% | 97.0% | 96.3% | 97.0% |
|  | 8 | 49.9% | 35.6% | 47.5% | 50.5% | 51.2% | 51.6% | 49.5% | 52.0% |
| 1g q24h post-HD | 2 | 99.2% | 99.2% | 99.2% | 99.2% | 99.2% | 99.2% | 99.0% | 99.2% |
|  | 8 | 87.9% | 84.7% | 87.7% | 88.0% | 88.0% | 88.0% | 86.3% | 88.0% |
| 0.5g q12h post-HD | 2 | 99.9% | 99.9% | 99.9% | 99.9% | 99.9% | 99.9% | 99.9% | 99.9% |
|  | 8 | 92.0% | 79.4% | 91.5% | 92.2% | 92.2% | 92.3% | 91.1% | 92.3% |
| 1g LD, 0.5g q12h post-HD | 2 | 99.9% | 99.9% | 99.9% | 99.9% | 99.9% | 99.9% | 99.9% | 99.9% |
|  | 8 | 93.8% | 97.5% | 92.4% | 92.2% | 92.2% | 92.2% | 91.0% | 92.2% |
| 1g q12h post-HD | 2 | 99.9% | 99.9% | 99.9% | 99.9% | 99.9% | 99.9% | 99.9% | 99.9% |
|  | 8 | 99.7% | 99.5% | 99.7% | 99.7% | 99.7% | 99.7% | 99.4% | 99.7% |

MIC: minimum inhibitory concentration; PTA: probability of target attainment; 40%*f*T> MIC: 40% of a dosing interval that a free drug concentration exceeds the MIC. All doses were simulated as a 30 min infusion.

The dose in yellow and green is the smallest meropenem dosing regimen attaining PD targets of 40% fT>MIC of 2 mg/L and 40% fT>MICx4 of 8mg/L respectively.

**Table S21. PTA of various meropenem dosing regimens in KRT setting 3 (Sequential therapy)**

| **Meropenem Dose** | **MIC**  **(mg/L)** | **PTA during 1-week of therapy** | | | | | | | |
| --- | --- | --- | --- | --- | --- | --- | --- | --- | --- |
|  |  | **Mean** | **Day 1** | **Day 2** | **Day 3** | **Day 4** | **Day 5** | **Day 6** | **Day 7** |
| 0.5g q24h post-HD | 2 | 96.9% | 96.8% | 96.9% | 96.9% | 96.9% | 96.9% | 96.1% | 96.9% |
|  | 8 | 6.8% | 29.0% | 40.7% | 43.6% | 44.5% | 44.8% | 42.1% | 45.0% |
| 1g q24h post-HD | 2 | 98.8% | 98.8% | 98.8% | 98.8% | 98.8% | 98.8% | 98.5% | 98.8% |
|  | 8 | 84.9% | 80.8% | 84.6% | 85.0% | 85.0% | 85.0% | 83.1% | 85.0% |
| 0.5g q12h post-HD | 2 | 99.9% | 99.9% | 99.9% | 99.9% | 99.9% | 99.9% | 99.9% | 99.9% |
|  | 8 | 89.9% | 75.5% | 89.1% | 90.2% | 90.4% | 90.4% | 88.8% | 90.5% |
| 1g LD, 0.5g q12h post-HD | 2 | 100.0% | 100.0% | 100.0% | 100.0% | 100.0% | 100.0% | 99.9% | 100.0% |
|  | 8 | 91.5% | 96.7% | 90.0% | 89.7% | 89.7% | 89.7% | 88.2% | 89.7% |
| 1g q12h post-HD | 2 | 100.0% | 100.0% | 100.0% | 100.0% | 100.0% | 100.0% | 100.0% | 100.0% |
|  | 8 | 99.6% | 99.4% | 99.6% | 99.6% | 99.6% | 99.6% | 99.2% | 99.6% |
| 0.5g q8h post-HD | 2 | 100.0% | 100.0% | 100.0% | 100.0% | 100.0% | 100.0% | 100.0% | 100.0% |
|  | 8 | 98.9% | 95.4% | 98.8% | 99.0% | 99.0% | 99.0% | 98.7% | 99.1% |

MIC: minimum inhibitory concentration; PTA: probability of target attainment; 40%*f*T> MIC: 40% of a dosing interval that a free drug concentration exceeds the MIC. All doses were simulated as a 30 min infusion.

The dose in yellow and green is the smallest meropenem dosing regimen attaining PD targets of 40% fT>MIC of 2 mg/L and 40% fT>MICx4 of 8mg/L respectively.

**Table S22. PTA of various meropenem dosing regimens in KRT setting 4 (Early 9-hour PIKRT)**

| **Meropenem Dose** | **MIC**  **(mg/L)** | **PTA during 1-week of therapy** | | | | | | | |
| --- | --- | --- | --- | --- | --- | --- | --- | --- | --- |
|  |  | **Mean** | **Day 1** | **Day 2** | **Day 3** | **Day 4** | **Day 5** | **Day 6** | **Day 7** |
| 0.5g q24h | 2 | 62.4% | 59.6% | 62.3% | 62.4% | 62.4% | 62.4% | 60.3% | 62.4% |
|  | 8 | 0.4% | 0.0% | 0.1% | 0.2% | 0.5% | 0.5% | 0.6% | 0.7% |
| 1g q24h | 2 | 81.5% | 81.4% | 81.5% | 81.5% | 81.5% | 81.5% | 80.2% | 81.5% |
|  | 8 | 21.8% | 6.4% | 19.1% | 22.1% | 22.8% | 22.9% | 21.7% | 23.0% |
| 0.5g q12h | 2 | 99.8% | 99.8% | 99.8% | 99.8% | 99.8% | 99.8% | 99.8% | 99.8% |
|  | 8 | 81.6% | 67.5% | 80.7% | 82.4% | 82.7% | 82.7% | 80.7% | 82.7% |
| 1g LD, 0.5g q12h | 2 | 99.8% | 99.9% | 99.8% | 99.8% | 99.8% | 99.8% | 99.7% | 99.8% |
|  | 8 | 83.5% | 87.7% | 81.5% | 81.6% | 81.6% | 81.6% | 79.6% | 81.6% |
| 1g q12h | 2 | 99.9% | 99.9% | 99.9% | 99.9% | 99.9% | 99.9% | 99.8% | 99.9% |
|  | 8 | 98.0% | 97.6% | 98.0% | 98.0% | 98.0% | 98.0% | 97.4% | 98.0% |
| 0.5g q8h | 2 | 99.9% | 99.9% | 99.9% | 99.9% | 99.9% | 99.9% | 99.9% | 99.9% |
|  | 8 | 97.8% | 91.6% | 97.8% | 97.8% | 97.8% | 97.8% | 97.1% | 97.9% |

MIC: minimum inhibitory concentration; PTA: probability of target attainment; 40%*f*T> MIC: 40% of a dosing interval that a free drug concentration exceeds the MIC. All doses were simulated as a 30 min infusion.

The dose in yellow and green is the smallest meropenem dosing regimen attaining PD targets of 40% fT>MIC of 2 mg/L and 40% fT>MICx4 of 8mg/L respectively.

**Table S23. PTA of various meropenem dosing regimens in KRT setting 5 (Late 9-hour PIKRT)**

| **Meropenem Dose** | **MIC**  **(mg/L)** | **PTA during 1-week of therapy** | | | | | | | |
| --- | --- | --- | --- | --- | --- | --- | --- | --- | --- |
|  |  | **Mean** | **Day 1** | **Day 2** | **Day 3** | **Day 4** | **Day 5** | **Day 6** | **Day 7** |
| 0.5g q24h post-PIKRT | 2 | 97.3% | 97.3% | 97.3% | 97.3% | 97.3% | 97.3% | 96.5% | 97.3% |
|  | 8 | 47.3% | 35.9% | 45.9% | 48.2% | 49.0% | 49.3% | 46.9% | 49.5% |
| 1g q24h post-PIKRT | 2 | 99.0% | 99.0% | 99.0% | 99.0% | 99.0% | 99.0% | 98.8% | 99.0% |
|  | 8 | 87.3% | 83.6% | 87.0% | 87.4% | 87.5% | 87.5% | 85.6% | 87.5% |
| 0.5g q12h post-PIKRT | 2 | 99.8% | 99.8% | 99.8% | 99.8% | 99.8% | 99.8% | 99.8% | 99.8% |
|  | 8 | 86.2% | 69.0% | 85.3% | 86.7% | 86.8% | 86.8% | 85.0% | 86.8% |
| 1g LD, 0.5g q12h post-PIKRT | 2 | 99.9% | 100.0% | 99.9% | 99.9% | 99.9% | 99.9% | 99.9% | 99.9% |
|  | 8 | 89.5% | 96.6% | 87.3% | 86.9% | 86.8% | 86.8% | 84.8% | 86.8% |
| 1g q12h post-PIKRT | 2 | 99.9% | 99.9% | 99.9% | 99.9% | 99.9% | 99.9% | 99.9% | 99.9% |
|  | 8 | 99.3% | 99.0% | 99.3% | 99.3% | 99.3% | 99.3% | 98.8% | 99.3% |
| 0.5g q8h post-PIKRT | 2 | 99.9% | 100.0% | 100.0% | 100.0% | 100.0% | 100.0% | 99.9% | 100.0% |
|  | 8 | 100.0% | 94.7% | 98.4% | 98.5% | 98.5% | 98.5% | 98.0% | 98.5% |

MIC: minimum inhibitory concentration; PTA: probability of target attainment; 40%*f*T> MIC: 40% of a dosing interval that a free drug concentration exceeds the MIC. All doses were simulated as a 30 min infusion.

The dose in yellow and green is the smallest meropenem dosing regimen attaining PD targets of 40% fT>MIC of 2 mg/L and 40% fT>MICx4 of 8mg/L respectively.

**Table S24. PTA of various meropenem dosing regimens in KRT setting 6 (Extended PIKRT)**

| **Meropenem Dose** | **MIC**  **(mg/L)** | **PTA during 1-week of therapy** | | | | | | | |
| --- | --- | --- | --- | --- | --- | --- | --- | --- | --- |
|  |  | **Mean** | **Day 1** | **Day 2** | **Day 3** | **Day 4** | **Day 5** | **Day 6** | **Day 7** |
| 0.5g q24h | 2 | 78.8% | 78.2% | 78.8% | 78.8% | 78.8% | 78.8% | 75.0% | 78.8% |
|  | 8 | 0.4% | 0.0% | 0.3% | 0.5% | 0.6% | 0.6% | 0.4% | 0.6% |
| 1g q24h | 2 | 91.6% | 91.6% | 91.6% | 91.6% | 91.6% | 91.6% | 89.7% | 91.6% |
|  | 8 | 38.4% | 27.9% | 37.5% | 38.7% | 39.0% | 39.0% | 33.9% | 39.1% |
| 0.5g q12h | 2 | 99.2% | 99.2% | 99.2% | 99.2% | 99.2% | 99.2% | 98.5% | 99.2% |
|  | 8 | 57.4% | 34.9% | 56.8% | 59.3% | 59.6% | 59.8% | 51.2% | 59.8% |
| 1g LD, 0.5g q12h | 2 | 99.5% | 99.8% | 99.5% | 99.5% | 99.5% | 99.5% | 98.8% | 99.5% |
|  | 8 | 60.9% | 79.5% | 59.4% | 59.1% | 59.1% | 59.2% | 50.7% | 59.2% |
| 1g q12h | 2 | 99.9% | 99.9% | 99.9% | 99.9% | 99.9% | 99.9% | 99.7% | 99.9% |
|  | 8 | 95.6% | 94.0% | 95.6% | 95.6% | 95.6% | 95.6% | 92.5% | 95.6% |

MIC: minimum inhibitory concentration; PTA: probability of target attainment; 40%*f*T> MIC: 40% of a dosing interval that a free drug concentration exceeds the MIC. All doses were simulated as a 30 min infusion.

The dose in yellow and green is the smallest meropenem dosing regimen attaining PD targets of 40% fT>MIC of 2 mg/L and 40% fT>MICx4 of 8mg/L respectively.

1. **Piperacillin/Tazobactam Simulation Results**

**Table S25. PTA of various piperacillin dosing regimens in KRT setting 1 (Thrice-weekly HD)**

| **Piperacillin Dose** | **MIC**  **(mg/L)** | **PTA during 1-week of therapy** | | | | | | | |
| --- | --- | --- | --- | --- | --- | --- | --- | --- | --- |
|  |  | **Mean** | **Day 1** | **Day 2** | **Day 3** | **Day 4** | **Day 5** | **Day 6** | **Day 7** |
| 2g q12h post-HD | 16 | 92.3% | 91.7% | 93.8% | 93.3% | 93.8% | 93.3% | 93.8% | 93.9% |
|  | 64 | 38.1% | 15.7% | 36.7% | 33.1% | 43.7% | 35.9% | 44.6% | 46.7% |
| 3g q12h post-HD | 16 | 96.4% | 97.0% | 97.4% | 97.3% | 97.4% | 97.3% | 97.4% | 97.4% |
|  | 64 | 61.7% | 39.3% | 62.0% | 59.7% | 66.0% | 61.0% | 66.3% | 67.1% |
| 4g q12h post-HD | 16 | 97.7% | 98.2% | 98.3% | 98.2% | 98.3% | 98.2% | 98.3% | 98.3% |
|  | 64 | 74.3% | 60.1% | 75.6% | 74.1% | 77.4% | 74.5% | 77.5% | 78.0% |
| 4g LD, 4g q12h EI post-HD | 16 | 99.7% | 99.0% | 99.8% | 99.8% | 99.8% | 99.8% | 99.8% | 99.8% |
|  | 64 | 79.0% | 56.6% | 79.4% | 78.2% | 81.9% | 78.9% | 82.0% | 82.2% |
| 2g q8h post-HD | 16 | 97.8% | 98.2% | 98.7% | 98.6% | 98.7% | 98.6% | 98.7% | 98.7% |
|  | 64 | 62.8% | 35.9% | 63.2% | 59.6% | 67.7% | 61.1% | 68.3% | 69.3% |
| 3g q8h post-HD | 16 | 99.4% | 99.7% | 99.7% | 99.7% | 99.7% | 99.7% | 99.7% | 99.7% |
|  | 64 | 81.6% | 65.8% | 83.6% | 81.1% | 85.0% | 81.4% | 85.1% | 85.5% |
| 3g LD, 3g q8h EI post-HD | 16 | 100.0% | 100.0% | 100.0% | 100.0% | 100.0% | 100.0% | 100.0% | 100.0% |
|  | 64 | 84.0% | 65.4% | 84.2% | 83.0% | 85.4% | 83.2% | 87.1% | 85.7% |
| 4g q8h post-HD | 16 | 99.6% | 99.8% | 99.8% | 99.8% | 99.8% | 99.8% | 99.8% | 99.8% |
|  | 64 | 89.4% | 82.0% | 90.9% | 89.7% | 91.4% | 89.7% | 91.4% | 91.5% |
| 4g LD, 4g q8h EI post-HD | 16 | 100.0% | 100.0% | 100.0% | 100.0% | 100.0% | 100.0% | 100.0% | 100.0% |
|  | 64 | 93.1% | 83.3% | 93.3% | 93.0% | 93.6% | 93.1% | 94.5% | 93.7% |
| 2g q6h post-HD | 16 | 99.6% | 99.9% | 99.6% | 99.9% | 99.6% | 99.9% | 99.9% | 99.6% |
|  | 64 | 76.9% | 57.7% | 76.4% | 75.8% | 78.5% | 76.2% | 80.9% | 79.0% |
| 2g LD, 2g q6h EI post-HD | 16 | 100.0% | 99.9% | 100.0% | 100.0% | 100.0% | 100.0% | 100.0% | 100.0% |
|  | 64 | 80.0% | 49.5% | 79.0% | 77.7% | 81.5% | 78.2% | 82.9% | 81.9% |
| 3g q6h post-HD | 16 | 99.8% | 100.0% | 99.8% | 100.0% | 99.8% | 100.0% | 100.0% | 99.8% |
|  | 64 | 91.7% | 83.1% | 91.9% | 91.8% | 92.1% | 91.9% | 93.8% | 92.1% |
| 3g LD, 3g q6h EI post-HD | 16 | 100.0% | 100.0% | 100.0% | 100.0% | 100.0% | 100.0% | 100.0% | 100.0% |
|  | 64 | 93.2% | 79.8% | 93.3% | 92.8% | 93.7% | 92.9% | 94.7% | 93.8% |
| 4g q6h post-HD | 16 | 99.9% | 100.0% | 99.9% | 100.0% | 99.9% | 100.0% | 100.0% | 99.9% |
|  | 64 | 95.1% | 92.9% | 95.2% | 95.6% | 95.2% | 95.7% | 96.7% | 95.2% |

MIC: minimum inhibitory concentration; PTA: probability of target attainment; 50%*f*T> MIC: 50% of a dosing interval that a free drug concentration exceeds the MIC. EI: 4-hour extended infusion. All other doses were simulated as a 30 min infusion.

The doses in yellow and green are the smallest piperacillin dosing regimens attaining PD targets of 50% fT>MIC of 16 mg/L and 50% fT>MICx4 of 64mg/L respectively.

**Table S26. PTA of various tazobactam dosing regimens in KRT setting 1 (Thrice-weekly HD)**

| **Tazobactam Dose**  (Accompanying Piperacillin Dose) | **PTA during 1-week of therapy** | | | | | | | |
| --- | --- | --- | --- | --- | --- | --- | --- | --- |
|  | **Mean** | **Day 1** | **Day 2** | **Day 3** | **Day 4** | **Day 5** | **Day 6** | **Day 7** |
| 0.25g q12h post-HD  (2g q12h post-HD) | 75.3% | 55.3% | 72.0% | 73.3% | 77.6% | 75.3% | 78.4% | 79.1% |
| 0.375g q12h post-HD  (3g q12h post-HD) | 83.9% | 73.0% | 83.0% | 83.6% | 85.5% | 84.0% | 85.6% | 85.8% |
| 0.5g q12h post-HD  (4g q12h post-HD) | 88.7% | 83.1% | 88.9% | 88.8% | 89.9% | 89.0% | 89.9% | 90.0% |
| 0.5g LD, 0.5g 12h EI post-HD  (4g LD, 4g q12h EI post-HD) | 91.3% | 76.8% | 91.1% | 91.3% | 92.4% | 91.6% | 92.4% | 92.5% |
| 0.25g q8h post-HD  (2g q8h post-HD) | 86.5% | 68.3% | 85.6% | 86.0% | 88.2% | 86.5% | 88.5% | 88.8% |
| 0.375g q8h post-HD  (3g q8h post-HD) | 91.5% | 83.9% | 91.9% | 91.6% | 92.7% | 91.9% | 92.7% | 92.8% |
| 0.375g LD, 0.375g q8h EI post-HD  (3g LD, 3g q8h EI post-HD) | 93.5% | 84.5% | 93.3% | 93.4% | 93.7% | 93.5% | 94.2% | 93.9% |
| 0.5g q8h post-HD  (4g q8h post-HD) | 94.0% | 90.3% | 94.7% | 94.4% | 94.9% | 94.5% | 94.9% | 95.0% |
| 0.5g LD, 0.5g q8h EI post-HD  (4g LD, 4g q8h EI post-HD) | 96.5% | 91.5% | 96.5% | 96.6% | 96.7% | 96.6% | 97.1% | 96.7% |
| 0.25g q6h post-HD  (2g q6h post-HD) | 90.3% | 80.3% | 89.4% | 90.3% | 90.6% | 90.6% | 91.9% | 90.8% |
| 0.25g LD, 0.25g q6h EI post-HD  (2g LD, 2g q6h EI) | 91.9% | 75.1% | 91.1% | 91.4% | 92.5% | 91.7% | 93.0% | 92.6% |
| 0.375g q6h post-HD  (3g q6h post-HD) | 94.8% | 91.2% | 94.6% | 95.1% | 94.9% | 95.2% | 95.8% | 95.0% |
| 0.375g LD, 0.375g q6h EI post-HD  (3g LD, 3g q6h EI post-HD) | 96.5% | 89.3% | 96.6% | 96.6% | 96.9% | 96.6% | 97.2% | 96.9% |
| 0.5g q6h post-HD  (4g q6h post-HD) | 96.6% | 95.6% | 96.6% | 97.1% | 96.7% | 97.1% | 97.4% | 96.7% |

The doses in yellow are the smallest tazobactam dosing regimens attaining the target of 50% fT>threshold of 4 mg/L.

**Table S27. PTA of various piperacillin dosing regimens in KRT setting 2 (Daily HD)**

| **Piperacillin Dose** | **MIC**  **(mg/L)** | **PTA during 1-week of therapy** | | | | | | | |
| --- | --- | --- | --- | --- | --- | --- | --- | --- | --- |
|  |  | **Mean** | **Day 1** | **Day 2** | **Day 3** | **Day 4** | **Day 5** | **Day 6** | **Day 7** |
| 2g q12h post-HD | 16 | 92.7% | 91.6% | 93.5% | 93.6% | 93.6% | 93.6% | 93.6% | 93.6% |
|  | 64 | 27.9% | 9.8% | 24.0% | 29.5% | 31.2% | 31.8% | 32.1% | 32.3% |
| 3g q12h post-HD | 16 | 95.9% | 96.4% | 96.7% | 96.7% | 96.7% | 96.7% | 96.7% | 96.7% |
|  | 64 | 57.1% | 32.4% | 55.3% | 58.9% | 59.8% | 60.2% | 60.3% | 60.3% |
| 4g q12h post-HD | 16 | 97.7% | 98.5% | 98.5% | 98.5% | 98.5% | 98.5% | 98.5% | 98.5% |
|  | 64 | 72.9% | 53.2% | 72.5% | 74.3% | 74.9% | 74.9% | 75.0% | 75.0% |
| 4g LD, 4g q12h EI post-HD | 16 | 99.5% | 99.0% | 99.8% | 99.8% | 99.8% | 99.8% | 99.8% | 99.8% |
|  | 64 | 75.3% | 53.4% | 74.3% | 76.9% | 77.5% | 77.7% | 77.7% | 77.7% |
| 2g q8h post-HD | 16 | 97.8% | 98.0% | 98.5% | 98.5% | 98.5% | 98.5% | 98.5% | 98.5% |
|  | 64 | 53.0% | 29.2% | 50.3% | 55.0% | 56.2% | 56.7% | 56.8% | 56.8% |
| 3g q8h post-HD | 16 | 99.4% | 99.7% | 99.7% | 99.7% | 99.7% | 99.7% | 99.7% | 99.7% |
|  | 64 | 78.3% | 61.7% | 78.0% | 79.8% | 80.1% | 80.3% | 80.3% | 80.3% |
| 3g LD, 3g q8h EI post-HD | 16 | 100.0% | 100.0% | 100.0% | 100.0% | 100.0% | 100.0% | 100.0% | 100.0% |
|  | 64 | 79.2% | 60.6% | 78.2% | 80.2% | 80.5% | 80.6% | 80.6% | 80.6% |
| 4g q8h post-HD | 16 | 99.7% | 99.8% | 99.8% | 99.8% | 99.8% | 99.8% | 99.8% | 99.8% |
|  | 64 | 87.5% | 79.3% | 88.3% | 88.8% | 88.9% | 88.9% | 88.9% | 88.9% |
| 4g LD, 4g q8h EI post-HD | 16 | 100.0% | 100.0% | 100.0% | 100.0% | 100.0% | 100.0% | 100.0% | 100.0% |
|  | 64 | 90.9% | 80.8% | 91.0% | 91.5% | 91.5% | 91.6% | 91.6% | 91.6% |
| 2g q6h post-HD | 16 | 99.6% | 99.6% | 99.6% | 99.6% | 99.6% | 99.6% | 99.6% | 99.6% |
|  | 64 | 73.2% | 50.0% | 72.0% | 74.7% | 75.1% | 75.3% | 75.3% | 75.3% |
| 2g LD, 2g q6h EI post-HD | 16 | 100.0% | 99.9% | 100.0% | 100.0% | 100.0% | 100.0% | 100.0% | 100.0% |
|  | 64 | 72.9% | 46.4% | 71.6% | 74.0% | 74.5% | 74.6% | 74.7% | 74.7% |
| 3g q6h post-HD | 16 | 99.7% | 99.7% | 99.7% | 99.7% | 99.7% | 99.7% | 99.7% | 99.7% |
|  | 64 | 88.8% | 79.8% | 88.9% | 89.6% | 89.8% | 89.8% | 89.8% | 89.8% |
| 3g LD, 3g q6h EI post-HD | 16 | 100.0% | 100.0% | 100.0% | 100.0% | 100.0% | 100.0% | 100.0% | 100.0% |
|  | 64 | 91.3% | 77.6% | 91.3% | 91.6% | 91.7% | 91.7% | 91.7% | 91.7% |
| 4g q6h post-HD | 16 | 100.0% | 100.0% | 100.0% | 100.0% | 100.0% | 100.0% | 100.0% | 100.0% |
|  | 64 | 94.7% | 91.4% | 95.0% | 95.1% | 95.2% | 95.2% | 95.2% | 95.2% |

MIC: minimum inhibitory concentration; PTA: probability of target attainment; 50%*f*T> MIC: 50% of a dosing interval that a free drug concentration exceeds the MIC. EI: 4-hour extended infusion. All other doses were simulated as a 30 min infusion.

The doses in yellow and green are the smallest piperacillin dosing regimens attaining PD targets of 50% fT>MIC of 16 mg/L and 50% fT>MICx4 of 64mg/L respectively.

**Table S28. PTA of various tazobactam dosing regimens in KRT setting 2 (Daily HD)**

| **Tazobactam Dose**  (Accompanying Piperacillin Dose) | **PTA during 1-week of therapy** | | | | | | | |
| --- | --- | --- | --- | --- | --- | --- | --- | --- |
|  | **Mean** | **Day 1** | **Day 2** | **Day 3** | **Day 4** | **Day 5** | **Day 6** | **Day 7** |
| 0.25g q12h post-HD  (2g q12h post-HD) | 71.2% | 43.7% | 65.4% | 70.9% | 72.7% | 73.3% | 73.6% | 73.9% |
| 0.375g q12h post-HD  (3g q12h post-HD) | 82.9% | 65.0% | 80.7% | 83.2% | 83.9% | 84.0% | 84.2% | 84.2% |
| 0.5g q12h post-HD  (4g q12h post-HD) | 88.0% | 78.6% | 87.2% | 88.5% | 88.7% | 88.8% | 88.8% | 88.8% |
| 0.5g LD, 0.5g q12h EI post-HD  (4g LD, 4g q12h EI post-HD) | 90.6% | 75.6% | 89.9% | 91.3% | 91.4% | 91.4% | 91.4% | 91.5% |
| 0.25g q8h post-HD  (2g q8h post-HD) | 83.7% | 66.1% | 81.5% | 84.3% | 85.0% | 85.1% | 85.2% | 85.3% |
| 0.375g q8h post-HD  (3g q8h post-HD) | 91.4% | 83.6% | 91.1% | 91.9% | 92.1% | 92.1% | 92.1% | 92.2% |
| 0.375g LD, 0.375g q8h EI post-HD  (3g LD, 3g q8h EI post-HD) | 92.6% | 82.8% | 92.2% | 93.0% | 93.2% | 93.2% | 93.2% | 93.2% |
| 0.5g q8h post-HD  (4g q8h post-HD) | 93.9% | 90.5% | 94.0% | 94.6% | 94.7% | 94.7% | 94.7% | 94.7% |
| 0.5g LD, 0.5g q8h EI post-HD  (4g LD, 4g q8h EI post-HD) | 95.4% | 91.0% | 95.4% | 95.8% | 95.8% | 95.8% | 95.8% | 95.8% |
| 0.25g q6h post-HD  (2g q6h post-HD) | 90.1% | 78.4% | 89.2% | 90.4% | 90.5% | 90.6% | 90.6% | 90.6% |
| 0.25g LD, 0.25g q6h EI post-HD  (2g LD, 2g q6h EI) | 92.6% | 77.2% | 91.9% | 92.7% | 92.9% | 92.9% | 92.9% | 92.9% |
| 0.375g q6h post-HD  (3g q6h post-HD) | 94.6% | 90.8% | 94.5% | 94.8% | 94.9% | 94.9% | 94.9% | 94.9% |
| 0.375g LD, 0.375g q6h EI post-HD  (3g LD, 3g q6h EI post-HD) | 96.7% | 91.2% | 96.6% | 96.8% | 96.8% | 96.8% | 96.8% | 96.8% |
| 0.5g q6h post-HD  (4g q6h post-HD) | 97.2% | 95.5% | 97.3% | 97.3% | 97.3% | 97.3% | 97.3% | 97.3% |

The doses in yellow are the smallest tazobactam dosing regimens attaining the target of 50% fT>threshold of 4 mg/L.

**Table S29. PTA of various piperacillin dosing regimens in KRT setting 3 (Sequential therapy)**

| **Piperacillin Dose** | **MIC**  **(mg/L)** | **PTA during 1-week of therapy** | | | | | | | |
| --- | --- | --- | --- | --- | --- | --- | --- | --- | --- |
|  |  | **Mean** | **Day 1** | **Day 2** | **Day 3** | **Day 4** | **Day 5** | **Day 6** | **Day 7** |
| 2g q12h post-HD | 16 | 91.1% | 89.4% | 92.0% | 92.2% | 92.2% | 92.2% | 92.2% | 92.2% |
|  | 64 | 20.4% | 7.5% | 18.1% | 22.2% | 23.6% | 24.4% | 24.7% | 24.8% |
| 3g q12h post-HD | 16 | 95.8% | 96.4% | 96.8% | 96.8% | 96.8% | 96.8% | 96.8% | 96.8% |
|  | 64 | 51.2% | 28.4% | 49.3% | 53.6% | 54.8% | 55.2% | 55.3% | 55.3% |
| 4g q12h post-HD | 16 | 97.6% | 98.1% | 98.2% | 98.2% | 98.2% | 98.2% | 98.2% | 98.2% |
|  | 64 | 69.5% | 50.6% | 69.3% | 71.3% | 71.9% | 71.9% | 71.9% | 72.0% |
| 4g LD, 4g q12h EI post-HD | 16 | 99.6% | 99.6% | 99.7% | 99.7% | 99.7% | 99.7% | 99.7% | 99.7% |
|  | 64 | 71.5% | 48.7% | 71.3% | 73.9% | 74.5% | 74.6% | 74.7% | 74.7% |
| 2g q8h post-HD | 16 | 97.3% | 97.6% | 98.3% | 98.3% | 98.3% | 98.3% | 98.3% | 98.3% |
|  | 64 | 48.2% | 24.7% | 46.1% | 50.2% | 51.6% | 51.2% | 51.8% | 52.2% |
| 3g q8h post-HD | 16 | 98.9% | 99.5% | 99.5% | 99.5% | 99.5% | 99.5% | 99.5% | 99.5% |
|  | 64 | 74.8% | 57.6% | 75.2% | 76.6% | 77.0% | 76.9% | 77.1% | 77.1% |
| 3g LD, 3g q8h EI post-HD | 16 | 100.0% | 99.9% | 100.0% | 100.0% | 100.0% | 100.0% | 100.0% | 100.0% |
|  | 64 | 76.8% | 57.7% | 76.2% | 78.0% | 78.3% | 78.3% | 78.4% | 78.3% |
| 4g q8h post-HD | 16 | 99.5% | 99.8% | 99.8% | 99.8% | 99.8% | 99.8% | 99.8% | 99.8% |
|  | 64 | 87.3% | 78.3% | 88.0% | 88.5% | 88.6% | 88.6% | 88.6% | 88.6% |
| 4g LD, 4g q8h EI post-HD | 16 | 100.0% | 100.0% | 100.0% | 100.0% | 100.0% | 100.0% | 100.0% | 100.0% |
|  | 64 | 89.4% | 78.3% | 89.3% | 90.1% | 90.2% | 90.1% | 90.2% | 90.2% |
| 2g q6h post-HD | 16 | 99.3% | 99.3% | 99.4% | 99.4% | 99.4% | 99.4% | 99.4% | 99.4% |
|  | 64 | 69.1% | 45.4% | 68.0% | 70.4% | 70.8% | 70.9% | 71.0% | 71.0% |
| 2g LD, 2g q6h EI post-HD | 16 | 100.0% | 99.9% | 100.0% | 100.0% | 100.0% | 100.0% | 100.0% | 100.0% |
|  | 64 | 71.5% | 43.9% | 65.2% | 72.5% | 73.3% | 73.3% | 73.5% | 73.5% |
| 3g q6h post-HD | 16 | 99.8% | 99.9% | 99.9% | 99.9% | 99.9% | 99.9% | 99.9% | 99.9% |
|  | 64 | 88.0% | 77.6% | 88.3% | 88.7% | 88.7% | 88.8% | 88.8% | 88.8% |
| 3g LD, 3g q6h EI post-HD | 16 | 100.0% | 100.0% | 100.0% | 100.0% | 100.0% | 100.0% | 100.0% | 100.0% |
|  | 64 | 89.9% | 74.9% | 87.6% | 90.7% | 91.0% | 91.1% | 91.2% | 91.2% |
| 4g q6h post-HD | 16 | 99.9% | 99.9% | 99.9% | 99.9% | 99.9% | 99.9% | 99.9% | 99.9% |
|  | 64 | 93.8% | 90.5% | 94.0% | 94.1% | 94.1% | 94.1% | 94.1% | 94.1% |

MIC: minimum inhibitory concentration; PTA: probability of target attainment; 50%*f*T> MIC: 50% of a dosing interval that a free drug concentration exceeds the MIC. EI: 4-hour extended infusion. All other doses were simulated as a 30 min infusion.

The doses in yellow and green are the smallest piperacillin dosing regimens attaining PD targets of 50% fT>MIC of 16 mg/L and 50% fT>MICx4 of 64mg/L respectively.

**Table S30. PTA of various tazobactam dosing regimens in KRT setting 3 (Sequential therapy)**

| **Tazobactam Dose**  (Accompanying Piperacillin Dose) | **PTA during 1-week of therapy** | | | | | | | |
| --- | --- | --- | --- | --- | --- | --- | --- | --- |
|  | **Mean** | **Day 1** | **Day 2** | **Day 3** | **Day 4** | **Day 5** | **Day 6** | **Day 7** |
| 0.25g q12h post-HD  (2g q12h post-HD) | 39.5% | 62.0% | 67.6% | 69.8% | 70.4% | 70.9% | 71.1% | 39.5% |
| 0.375g q12h post-HD  (3g q12h post-HD) | 81.8% | 63.0% | 80.0% | 82.4% | 83.1% | 83.3% | 83.4% | 83.5% |
| 0.5g q12h post-HD  (4g q12h post-HD) | 87.4% | 76.8% | 86.7% | 87.8% | 88.1% | 88.1% | 88.1% | 88.2% |
| 0.5g LD, 0.5g q12h EI post-HD  (4g LD, 4g q12h EI post-HD) | 89.3% | 75.1% | 88.5% | 89.9% | 90.2% | 90.4% | 90.4% | 90.4% |
| 0.25g q8h post-HD  (2g q8h post-HD) | 82.3% | 64.5% | 79.9% | 82.8% | 83.3% | 83.5% | 83.6% | 83.5% |
| 0.375g q8h post-HD  (3g q8h post-HD) | 90.1% | 81.3% | 89.4% | 90.7% | 90.9% | 90.9% | 90.9% | 91.0% |
| 0.375g LD, 0.375g q8h EI post-HD  (3g LD, 3g q8h EI post-HD) | 92.3% | 82.4% | 91.8% | 92.5% | 92.6% | 92.6% | 92.6% | 92.6% |
| 0.5g q8h post-HD  (4g q8h post-HD) | 93.6% | 89.3% | 93.6% | 94.5% | 94.6% | 94.6% | 94.6% | 94.6% |
| 0.5g LD, 0.5g q8h EI post-HD  (4g LD, 4g q8h EI post-HD) | 96.0% | 90.7% | 95.9% | 96.2% | 96.2% | 96.2% | 96.2% | 96.2% |
| 0.25g q6h post-HD  (2g q6h post-HD) | 89.0% | 77.1% | 88.1% | 89.3% | 89.5% | 89.5% | 89.5% | 89.5% |
| 0.25g LD, 0.25g q6h EI post-HD  (2g LD, 2g q6h EI) | 89.8% | 71.1% | 86.7% | 90.1% | 90.6% | 90.7% | 90.7% | 90.7% |
| 0.375g q6h post-HD  (3g q6h post-HD) | 94.7% | 90.5% | 94.7% | 95.0% | 95.0% | 95.0% | 95.0% | 95.0% |
| 0.375g LD, 0.375g q6h EI post-HD  (3g LD, 3g q6h EI post-HD) | 95.9% | 88.4% | 95.0% | 96.2% | 96.3% | 96.3% | 96.3% | 96.3% |
| 0.5g q6h post-HD  (4g q6h post-HD) | 96.2% | 94.3% | 96.3% | 96.3% | 96.3% | 96.3% | 96.3% | 96.3% |

The doses in yellow are the smallest tazobactam dosing regimens attaining the target of 50% fT>threshold of 4 mg/L.

**Table S31. PTA of various piperacillin dosing regimens in KRT setting 4 (Early 9-hour PIKRT)**

| **Piperacillin Dose** | **MIC**  **(mg/L)** | **PTA during 1-week of therapy** | | | | | | | |
| --- | --- | --- | --- | --- | --- | --- | --- | --- | --- |
|  |  | **Mean** | **Day 1** | **Day 2** | **Day 3** | **Day 4** | **Day 5** | **Day 6** | **Day 7** |
| 2g q12h | 16 | 88.1% | 86.2% | 89.3% | 89.4% | 89.4% | 89.4% | 89.4% | 89.4% |
|  | 64 | 0.0% | 5.1% | 12.8% | 16.4% | 17.8% | 18.6% | 19.0% | 19.1% |
| 3g q12h | 16 | 93.8% | 94.0% | 94.5% | 94.5% | 94.5% | 94.5% | 94.5% | 94.5% |
|  | 64 | 41.9% | 23.4% | 40.9% | 44.4% | 45.3% | 45.7% | 45.9% | 46.0% |
| 4g q12h | 16 | 95.9% | 96.3% | 96.4% | 96.4% | 96.4% | 96.4% | 96.4% | 96.4% |
|  | 64 | 60.7% | 45.1% | 60.9% | 63.2% | 63.6% | 63.7% | 63.7% | 63.8% |
| 4g LD, 4g q12h EI | 16 | 99.0% | 88.5% | 99.4% | 99.4% | 99.4% | 99.4% | 99.4% | 99.4% |
|  | 64 | 66.4% | 4.6% | 63.9% | 69.2% | 70.0% | 70.1% | 70.2% | 70.2% |
| 2g q8h | 16 | 96.3% | 96.1% | 96.8% | 96.8% | 96.8% | 96.8% | 96.8% | 96.8% |
|  | 64 | 45.4% | 17.8% | 42.3% | 47.6% | 48.6% | 48.9% | 49.0% | 49.0% |
| 3g q8h | 16 | 98.9% | 99.0% | 99.0% | 99.0% | 99.0% | 99.0% | 99.0% | 99.0% |
|  | 64 | 72.4% | 51.0% | 72.9% | 74.3% | 74.5% | 74.5% | 74.6% | 74.6% |
| 4g q8h | 16 | 99.5% | 99.5% | 99.5% | 99.5% | 99.5% | 99.5% | 99.5% | 99.5% |
|  | 64 | 86.1% | 73.7% | 86.8% | 87.2% | 87.2% | 87.2% | 87.2% | 87.2% |
| 4g LD, 4g q8h EI | 16 | 100.0% | 100.0% | 100.0% | 100.0% | 100.0% | 100.0% | 100.0% | 100.0% |
|  | 64 | 90.1% | 75.5% | 90.3% | 91.0% | 91.0% | 91.1% | 91.1% | 91.1% |
| 2g q6h | 16 | 98.9% | 99.1% | 99.2% | 99.2% | 99.2% | 99.2% | 99.2% | 99.2% |
|  | 64 | 65.5% | 40.1% | 64.8% | 67.9% | 68.6% | 68.7% | 68.7% | 68.7% |
| 3g q6h | 16 | 99.6% | 99.8% | 99.8% | 99.8% | 99.8% | 99.8% | 99.8% | 99.8% |
|  | 64 | 85.9% | 74.7% | 86.8% | 87.3% | 87.3% | 87.3% | 87.3% | 87.3% |
| 3g LD, 3g q6h EI | 16 | 100.0% | 100.0% | 100.0% | 100.0% | 100.0% | 100.0% | 100.0% | 100.0% |
|  | 64 | 91.6% | 72.3% | 91.2% | 92.0% | 92.1% | 92.1% | 92.1% | 92.1% |
| 4g q6h | 16 | 99.9% | 99.9% | 99.9% | 99.9% | 99.9% | 99.9% | 99.9% | 99.9% |
|  | 64 | 93.7% | 90.1% | 94.3% | 94.5% | 94.5% | 94.5% | 94.5% | 94.5% |
| 4g LD, 4g q6h EI | 16 | 100.0% | 100.0% | 100.0% | 100.0% | 100.0% | 100.0% | 100.0% | 100.0% |
|  | 64 | 97.2% | 89.1% | 97.4% | 97.4% | 97.4% | 97.4% | 97.4% | 97.4% |

MIC: minimum inhibitory concentration; PTA: probability of target attainment; 50%*f*T> MIC: 50% of a dosing interval that a free drug concentration exceeds the MIC. EI: 4-hour extended infusion. All other doses were simulated as a 30 min infusion.

The doses in yellow and green are the smallest piperacillin dosing regimens attaining PD targets of 50% fT>MIC of 16 mg/L and 50% fT>MICx4 of 64mg/L respectively.

**Table S32. PTA of various tazobactam dosing regimens in KRT setting 4 (Early 9-hour PIKRT)**

| **Tazobactam Dose**  (Accompanying Piperacillin Dose) | **PTA during 1-week of therapy** | | | | | | | |
| --- | --- | --- | --- | --- | --- | --- | --- | --- |
|  | **Mean** | **Day 1** | **Day 2** | **Day 3** | **Day 4** | **Day 5** | **Day 6** | **Day 7** |
| 0.25g q12h  (2g q12h) | 63.4% | 41.4% | 59.0% | 64.3% | 66.1% | 66.9% | 67.2% | 67.3% |
| 0.375g q12h  (3g q12h) | 77.3% | 64.3% | 76.5% | 78.3% | 78.9% | 79.0% | 79.0% | 79.1% |
| 0.5g q12h  (4g q12h) | 85.2% | 77.6% | 85.0% | 85.8% | 86.0% | 86.0% | 86.1% | 86.1% |
| 0.5g LD, 0.5g q12h EI  (4g LD, 4g q12h EI) | 87.9% | 70.3% | 87.7% | 88.6% | 88.8% | 89.0% | 89.0% | 89.0% |
| 0.25g q8h  (2g q8h) | 80.9% | 56.0% | 78.1% | 80.8% | 81.7% | 81.9% | 82.4% | 82.5% |
| 0.375g q8h  (3g q8h) | 89.7% | 77.3% | 89.2% | 90.0% | 90.2% | 90.2% | 90.5% | 90.5% |
| 0.5g q8h  (4g q8h) | 93.0% | 87.1% | 93.0% | 93.3% | 93.3% | 93.3% | 93.5% | 93.4% |
| 0.5g LD, 0.5g q8h EI  (4g LD, 4g q8h EI) | 95.0% | 88.2% | 95.1% | 95.3% | 95.4% | 95.5% | 95.5% | 95.5% |
| 0.25g q6h  (2g q6h) | 87.1% | 74.4% | 86.4% | 87.7% | 88.0% | 88.1% | 88.0% | 88.1% |
| 0.375g q6h  (3g q6h) | 93.4% | 88.4% | 93.6% | 93.9% | 94.0% | 94.0% | 93.9% | 94.0% |
| 0.375g LD, 0.375g q6h EI  (3g LD, 3g q6h EI) | 95.8% | 84.4% | 95.4% | 95.9% | 96.0% | 96.0% | 96.0% | 96.0% |
| 0.5g q6h  (4g q6h) | 96.0% | 93.9% | 96.3% | 96.4% | 96.4% | 96.4% | 96.4% | 96.4% |
| 0.5g LD, 0.5 q6h EI  (4g LD, 4g q6h EI) | 98.2% | 93.2% | 98.2% | 98.3% | 98.3% | 98.3% | 98.3% | 98.3% |

The doses in yellow are the smallest tazobactam dosing regimens attaining the target of 50% fT>threshold of 4 mg/L.

**Table S33. PTA of various piperacillin dosing regimens in KRT setting 5 (Late 9-hour PIKRT)**

| **Piperacillin Dose** | **MIC**  **(mg/L)** | **PTA during 1-week of therapy** | | | | | | | |
| --- | --- | --- | --- | --- | --- | --- | --- | --- | --- |
|  |  | **Mean** | **Day 1** | **Day 2** | **Day 3** | **Day 4** | **Day 5** | **Day 6** | **Day 7** |
| 2g q12h | 16 | 89.9% | 88.2% | 90.7% | 90.7% | 90.8% | 90.8% | 90.8% | 90.8% |
|  | 64 | 18.8% | 5.9% | 15.8% | 19.8% | 21.6% | 22.3% | 22.6% | 22.7% |
| 3g q12h | 16 | 94.9% | 95.2% | 95.6% | 95.6% | 95.6% | 95.6% | 95.6% | 95.6% |
|  | 64 | 48.5% | 24.4% | 46.5% | 50.9% | 52.0% | 52.3% | 52.5% | 52.6% |
| 4g q12h | 16 | 96.5% | 97.0% | 97.1% | 97.1% | 97.1% | 97.1% | 97.1% | 97.1% |
|  | 64 | 65.6% | 45.8% | 65.4% | 67.7% | 68.3% | 68.5% | 68.5% | 68.5% |
| 4g LD, 4g q12h EI | 16 | 99.4% | 98.3% | 99.7% | 99.7% | 99.7% | 99.7% | 99.7% | 99.7% |
|  | 64 | 67.6% | 45.5% | 66.8% | 69.9% | 70.4% | 70.6% | 70.7% | 70.7% |
| 2g q8h | 16 | 98.1% | 97.9% | 98.5% | 98.5% | 98.5% | 98.5% | 98.5% | 98.5% |
|  | 64 | 48.6% | 20.1% | 45.1% | 49.9% | 51.3% | 51.9% | 52.1% | 51.8% |
| 3g q8h | 16 | 99.6% | 99.7% | 99.7% | 99.7% | 99.7% | 99.7% | 99.7% | 99.7% |
|  | 64 | 76.5% | 56.1% | 76.2% | 77.9% | 78.3% | 78.4% | 78.5% | 78.4% |
| 4g q8h | 16 | 99.9% | 99.9% | 99.9% | 99.9% | 99.9% | 99.9% | 99.9% | 99.9% |
|  | 64 | 86.5% | 75.7% | 86.7% | 87.4% | 87.4% | 87.4% | 87.5% | 87.4% |
| 4g LD, 4g q8h EI | 16 | 100.0% | 100.0% | 100.0% | 100.0% | 100.0% | 100.0% | 100.0% | 100.0% |
|  | 64 | 90.3% | 79.4% | 90.6% | 91.2% | 91.2% | 91.2% | 91.2% | 91.2% |
| 2g q6h | 16 | 99.6% | 99.6% | 99.6% | 99.6% | 99.6% | 99.6% | 99.6% | 99.6% |
|  | 64 | 68.6% | 44.0% | 67.3% | 70.0% | 70.9% | 70.8% | 70.9% | 70.9% |
| 3g q6h | 16 | 99.9% | 99.9% | 99.9% | 99.9% | 99.9% | 99.9% | 99.9% | 99.9% |
|  | 64 | 87.6% | 77.3% | 88.4% | 89.0% | 89.1% | 89.0% | 89.0% | 89.0% |
| 3g LD, 3g q6h EI | 16 | 100.0% | 100.0% | 100.0% | 100.0% | 100.0% | 100.0% | 100.0% | 100.0% |
|  | 64 | 90.0% | 75.9% | 89.8% | 90.6% | 90.6% | 90.6% | 90.7% | 90.7% |
| 4g q6h | 16 | 99.9% | 99.9% | 99.9% | 99.9% | 99.9% | 99.9% | 99.9% | 99.9% |
|  | 64 | 94.6% | 90.4% | 95.0% | 95.1% | 95.2% | 95.1% | 95.1% | 95.1% |
| 4g LD, 4g q6h EI | 16 | 100.0% | 100.0% | 100.0% | 100.0% | 100.0% | 100.0% | 100.0% | 100.0% |
|  | 64 | 97.0% | 90.5% | 97.0% | 97.1% | 97.1% | 97.1% | 97.1% | 97.1% |

MIC: minimum inhibitory concentration; PTA: probability of target attainment; 50%*f*T> MIC: 50% of a dosing interval that a free drug concentration exceeds the MIC. EI: 4-hour extended infusion. All other doses were simulated as a 30 min infusion.

The doses in yellow and green are the smallest piperacillin dosing regimens attaining PD targets of 50% fT>MIC of 16 mg/L and 50% fT>MICx4 of 64mg/L respectively.

**Table S34. PTA of various tazobactam dosing regimens in KRT setting 5 (Late 9-hour PIKRT)**

| **Tazobactam Dose**  (Accompanying Piperacillin Dose) | **PTA during 1-week of therapy** | | | | | | | |
| --- | --- | --- | --- | --- | --- | --- | --- | --- |
|  | **Mean** | **Day 1** | **Day 2** | **Day 3** | **Day 4** | **Day 5** | **Day 6** | **Day 7** |
| 0.25g q12h  (2g q12h) | 66.7% | 37.3% | 60.7% | 66.8% | 68.7% | 69.3% | 69.6% | 69.7% |
| 0.375g q12h  (3g q12h) | 80.3% | 61.6% | 78.2% | 81.0% | 81.7% | 82.0% | 82.1% | 82.2% |
| 0.5g q12h  (4g q12h) | 87.3% | 77.1% | 87.0% | 88.0% | 88.2% | 88.2% | 88.2% | 88.2% |
| 0.5g LD, 0.5g q12h EI  (4g LD, 4g q12h EI) | 88.8% | 74.3% | 87.8% | 89.3% | 89.7% | 89.8% | 89.8% | 89.8% |
| 0.25g q8h  (2g q8h) | 82.4% | 63.8% | 80.4% | 82.8% | 83.6% | 83.7% | 83.8% | 83.8% |
| 0.375g q8h  (3g q8h) | 89.7% | 80.6% | 89.2% | 90.1% | 90.3% | 90.4% | 90.4% | 90.4% |
| 0.5g q8h  (4g q8h) | 93.9% | 89.8% | 94.2% | 94.5% | 94.5% | 94.5% | 94.5% | 94.5% |
| 0.5g LD, 0.5g q8h EI  (4g LD, 4g q8h EI) | 95.6% | 90.8% | 95.6% | 95.8% | 95.8% | 95.8% | 95.8% | 95.8% |
| 0.25g q6h  (2g q6h) | 88.9% | 75.8% | 88.1% | 89.4% | 89.6% | 89.7% | 89.8% | 89.8% |
| 0.375g q6h  (3g q6h) | 94.4% | 89.5% | 94.4% | 94.6% | 94.7% | 94.7% | 94.7% | 94.7% |
| 0.375g LD, 0.375g q6h EI  (3g LD, 3g q6h EI) | 95.9% | 88.3% | 95.7% | 96.0% | 96.1% | 96.1% | 96.1% | 96.1% |
| 0.5g q6h  (4g q6h) | 96.5% | 94.9% | 96.6% | 96.6% | 96.6% | 96.6% | 96.6% | 96.6% |
| 0.5g LD, 0.5 q6h EI  (4g LD, 4g q6h EI) | 97.6% | 93.7% | 97.6% | 97.7% | 97.7% | 97.7% | 97.7% | 97.7% |

The doses in yellow are the smallest tazobactam dosing regimens attaining the target of 50% fT>threshold of 4 mg/L.

**Table S35. PTA of various piperacillin dosing regimens in KRT setting 6 (Extended PIKRT)**

| **Piperacillin Dose** | **MIC**  **(mg/L)** | **PTA during 1-week of therapy** | | | | | | | |
| --- | --- | --- | --- | --- | --- | --- | --- | --- | --- |
|  |  | **Mean** | **Day 1** | **Day 2** | **Day 3** | **Day 4** | **Day 5** | **Day 6** | **Day 7** |
| 2g q12h | 16 | 81.9% | 80.2% | 85.1% | 85.2% | 85.2% | 85.2% | 85.2% | 85.2% |
|  | 64 | 1.9% | 0.1% | 1.6% | 2.7% | 3.3% | 3.5% | 3.6% | 3.7% |
| 3g q12h | 16 | 90.5% | 91.6% | 92.5% | 92.5% | 92.5% | 92.5% | 92.5% | 92.5% |
|  | 64 | 19.6% | 6.1% | 20.1% | 23.6% | 24.8% | 25.3% | 25.5% | 25.5% |
| 4g q12h | 16 | 94.0% | 95.4% | 95.5% | 95.5% | 95.5% | 95.5% | 95.5% | 95.5% |
|  | 64 | 42.6% | 23.7% | 44.7% | 48.3% | 48.9% | 49.2% | 49.2% | 49.2% |
| 2g q8h | 16 | 95.4% | 96.1% | 97.1% | 97.1% | 97.1% | 97.1% | 97.1% | 97.1% |
|  | 64 | 22.1% | 5.5% | 22.3% | 26.8% | 27.9% | 28.5% | 28.7% | 28.9% |
| 3g q8h | 16 | 98.1% | 98.9% | 98.9% | 98.9% | 98.9% | 98.9% | 98.9% | 98.9% |
|  | 64 | 58.5% | 35.7% | 61.4% | 64.3% | 64.9% | 64.9% | 65.0% | 65.0% |
| 3g LD, 3g q8h EI | 16 | 100.0% | 99.9% | 100.0% | 100.0% | 100.0% | 100.0% | 100.0% | 100.0% |
|  | 64 | 65.7% | 36.0% | 66.1% | 69.4% | 70.2% | 70.3% | 70.4% | 70.4% |
| 4g q8h | 16 | 99.0% | 99.5% | 99.5% | 99.5% | 99.5% | 99.5% | 99.5% | 99.5% |
|  | 64 | 75.9% | 64.1% | 79.5% | 80.3% | 80.5% | 80.5% | 80.6% | 80.6% |
| 4g LD, 4g q8h EI | 16 | 100.0% | 100.0% | 100.0% | 100.0% | 100.0% | 100.0% | 100.0% | 100.0% |
|  | 64 | 84.1% | 65.8% | 85.9% | 86.9% | 87.0% | 87.1% | 87.1% | 87.1% |
| 2g q6h | 16 | 98.6% | 99.3% | 99.5% | 99.5% | 99.5% | 99.5% | 99.5% | 99.5% |
|  | 64 | 51.3% | 24.1% | 53.7% | 57.6% | 58.5% | 58.7% | 58.8% | 58.9% |
| 3g q6h | 16 | 99.4% | 99.7% | 99.7% | 99.7% | 99.7% | 99.7% | 99.7% | 99.7% |
|  | 64 | 77.6% | 64.3% | 82.2% | 83.3% | 83.4% | 83.4% | 83.4% | 83.4% |
| 3g LD, 3g q6h EI | 16 | 100.0% | 100.0% | 100.0% | 100.0% | 100.0% | 100.0% | 100.0% | 100.0% |
|  | 64 | 84.9% | 62.3% | 85.7% | 87.0% | 87.1% | 87.1% | 87.2% | 87.2% |
| 4g q6h | 16 | 99.6% | 99.9% | 99.9% | 99.9% | 99.9% | 99.9% | 99.9% | 99.9% |
|  | 64 | 89.6% | 85.0% | 92.3% | 92.4% | 92.5% | 92.5% | 92.5% | 92.5% |
| 4g LD, 4g q6h EI | 16 | 100.0% | 100.0% | 100.0% | 100.0% | 100.0% | 100.0% | 100.0% | 100.0% |
|  | 64 | 94.6% | 85.3% | 95.6% | 95.7% | 95.7% | 95.7% | 95.7% | 95.7% |

MIC: minimum inhibitory concentration; PTA: probability of target attainment; 50%*f*T> MIC: 50% of a dosing interval that a free drug concentration exceeds the MIC. EI: 4-hour extended infusion. All other doses were simulated as a 30 min infusion.

The doses in yellow and green are the smallest piperacillin dosing regimens attaining PD targets of 50% fT>MIC of 16 mg/L and 50% fT>MICx4 of 64mg/L respectively.

**Table S36. PTA of various tazobactam dosing regimens in KRT setting 6 (Extended PIKRT)**

| **Tazobactam Dose**  (Accompanying Piperacillin Dose) | **PTA during 1-week of therapy** | | | | | | | |
| --- | --- | --- | --- | --- | --- | --- | --- | --- |
|  | **Mean** | **Day 1** | **Day 2** | **Day 3** | **Day 4** | **Day 5** | **Day 6** | **Day 7** |
| 0.25g q12h  (2g q12h) | 41.4% | 19.5% | 39.7% | 44.7% | 46.7% | 47.3% | 47.6% | 47.8% |
| 0.375g q12h  (3g q12h) | 66.4% | 47.9% | 66.3% | 69.6% | 70.4% | 70.7% | 70.9% | 70.9% |
| 0.5g q12h  (4g q12h) | 78.8% | 67.7% | 79.4% | 80.9% | 81.2% | 81.2% | 81.2% | 81.3% |
| 0.25g q8h  (2g q8h) | 69.4% | 47.1% | 68.1% | 71.8% | 73.0% | 73.5% | 73.7% | 73.8% |
| 0.375g q8h  (3g q8h) | 84.5% | 73.6% | 85.5% | 86.7% | 86.9% | 87.0% | 87.0% | 87.0% |
| 0.375g LD, 0.375g q8h EI  (3g LD, 3g q8h EI) | 88.4% | 73.7% | 88.4% | 89.7% | 90.0% | 90.1% | 90.1% | 90.1% |
| 0.5g q8h  (4g q8h) | 89.8% | 84.7% | 91.5% | 91.8% | 91.9% | 91.9% | 91.9% | 91.9% |
| 0.5g LD, 0.5g q8h EI  (4g LD, 4g q8h EI) | 93.8% | 85.8% | 94.7% | 95.0% | 95.0% | 95.0% | 95.0% | 95.0% |
| 0.25g q6h  (2g q6h) | 82.1% | 65.7% | 82.8% | 84.8% | 85.0% | 85.1% | 85.2% | 85.2% |
| 0.375g q6h  (3g q6h) | 91.0% | 85.1% | 92.7% | 93.2% | 93.2% | 93.2% | 93.2% | 93.2% |
| 0.375g LD, 0.375g q6h EI  (3g LD, 3g q6h EI) | 94.1% | 83.4% | 94.1% | 94.8% | 94.9% | 94.9% | 94.9% | 94.9% |
| 0.5g q6h  (4g q6h) | 95.1% | 93.2% | 96.2% | 96.3% | 96.3% | 96.3% | 96.3% | 96.3% |
| 0.5g LD, 0.5 q6h EI  (4g LD, 4g q6h EI) | 97.1% | 92.5% | 97.5% | 97.6% | 97.6% | 97.6% | 97.6% | 97.6% |

The doses in yellow are the smallest tazobactam dosing regimens attaining the target of 50% fT>threshold of 4 mg/L.

**Table S37. Probability of Neurotoxicity of MCS-driven Ceftazidime Dosing Recommendation in Five Kidney Replacement Therapies.**

|  | KRT setting | PD target^†^ | MCS-driven Ceftazidime dosing recommendation | Probability of total concentration above neurotoxicity threshold at the end of each day during 1 week of therapy^£^ | | | | | | |
| --- | --- | --- | --- | --- | --- | --- | --- | --- | --- | --- |
|  |  |  |  | Day 1 | Day 2 | Day 3 | Day 4 | Day 5 | Day 6 | Day 7 |
| 1 | 4-hour HD on Mon-Wed-Fri | 60% fT>MIC | 1g q24h post-HD | 0.0% | 0.0% | 0.0% | 0.0% | 0.0% | 0.0% | 0.0% |
|  |  | 60% fT>MICx4 | 2g LD, 1g q8h post-HD | 24.6% | 0.0% | 26.0% | 0.1% | 28.3% | 52.0% | 1.3% |
| 2 | 4-hour HD daily | 60% fT>MIC | 1g q24h post-HD | 0.0% | 0.0% | 0.0% | 0.0% | 0.0% | 0.0% | 0.0% |
|  |  | 60% fT>MICx4 | 2g LD, 1g q8h post-HD | 0.0% | 0.0% | 0.0% | 0.0% | 0.0% | 0.0% | 0.0% |
| 3 | Sequential 4-hour HD & 20-hour UF | 60% fT>MIC | 1g q24h post-HD | 0.0% | 0.0% | 0.0% | 0.0% | 0.0% | 0.0% | 0.0% |
|  |  | 60% fT>MICx4 | 2g LD, 1g q8h post-HD | 0.0% | 0.0% | 0.0% | 0.0% | 0.0% | 0.0% | 0.0% |
| 4 | Early 9-hour  PIKRT^€^  daily | 60% fT>MIC | 1g q12h | 0.0% | 0.0% | 0.0% | 0.0% | 0.0% | 0.0% | 0.0% |
|  |  | 60% fT>MICx4 | 2g LD, 1g q8h | 1.0% | 1.4% | 1.5% | 1.6% | 1.6% | 2.2% | 2.4% |
|  | Late 9-hour  PIKRT^€^ daily | 60% fT>MIC | 1g q12h | 0.0% | 0.0% | 0.0% | 0.0% | 0.0% | 0.0% | 0.0% |
|  |  | 60% fT>MICx4 | 2g LD, 1g q8h | 0.0% | 0.0% | 0.0% | 0.0% | 0.0% | 0.0% | 0.0% |
| 5 | Extended PIKRT daily | 60% fT>MIC | 1g q12h | 0.0% | 0.0% | 0.0% | 0.0% | 0.0% | 0.0% | 0.0% |
|  |  | 60% fT>MICx4 | 2g q8h | 0.0% | 0.6% | 1.4% | 1.9% | 2.1% | 3.5% | 2.5% |

KRT: kidney replacement therapy; PD: pharmacodynamic; MCS: Monte carlo simulation; HD: Hemodialysis at dialysate flow rate (Qd) 300 ml/min; UF: Ultrafiltration at ultrafilration flow rate of 5 ml/min; PIKRT: Prolonged intermittent kidney replacement therapy (9-hour PIKRT runs at Qd 100 ml/minl and extended PIKRT runs at Qd 50 ml/min for 24 hours); LD: loading dose

^†^60% fT>MIC or 60% fT>MICx4 denotes at least 60% of time during each day of one week of antibiotic therapy that the free plasma ceftazidime concentration was greater than the target minimum inhibitory concentration (MIC) or four time of the target MIC of 8 mg/L (susceptibility breakpoint MIC for *P. aeruginosa*).

^£^This indicates the percentage of 5000 simulated patients that were at or above the suggested ceftazidime safety threshold (i.e. free plasma concentration ≥ MICx8 = 64 mg/L) at the end of each day during 1 week of therapy.

^€^ EARLY PIKRT is where the initial ceftazidime dose is infused at the beginning of 9-hour PIKRT and late PIKRT where the initial ceftazidime dose is given 15 hours prior to 9-hour PIKRT virtual patients receiving each of 5 kidney replacement therapy.

**Table S38. Probability of Neurotoxicity of MCS-driven Imipenem Dosing Recommendation in Five Kidney Replacement Therapies.**

|  | KRT setting | PD target^†^ | MCS-driven Imipenem dosing recommendation | Probability of total concentration above neurotoxicity threshold at the end of each day during 1 week of therapy^£^ | | | | | | |
| --- | --- | --- | --- | --- | --- | --- | --- | --- | --- | --- |
|  |  |  |  | Day 1 | Day 2 | Day 3 | Day 4 | Day 5 | Day 6 | Day 7 |
| 1 | 4-hour HD on Mon-Wed-Fri | 40% fT>MIC | 500 mg q12h post-HD | 0.0% | 0.0% | 0.0% | 0.0% | 0.0% | 0.0% | 0.0% |
|  |  | 40% fT>MICx4 | 750 mg q8h post-HD | 3.6% | 0.3% | 5.9% | 0.5% | 5.9% | 8.3% | 0.7% |
| 2 | 4-hour HD daily | 40% fT>MIC | 500 mg q12h post-HD | 0.0% | 0.0% | 0.0% | 0.0% | 0.0% | 0.0% | 0.0% |
|  |  | 40% fT>MICx4 | 750 mg q8h post-HD | 0.0% | 0.0% | 0.2% | 0.2% | 0.2% | 0.4% | 0.2% |
| 3 | Sequential 4-hour HD & 20-hour UF | 40% fT>MIC | 500 mg q12h post-HD | 0.0% | 0.0% | 0.0% | 0.0% | 0.0% | 0.0% | 0.0% |
|  |  | 40% fT>MICx4 | 750 mg q8h post-HD | 0.0% | 0.0% | 0.1% | 0.1% | 0.1% | 0.2% | 0.1% |
| 4 | Early 9-hour  PIKRT^€^  daily | 40% fT>MIC | 500 mg q12h | 0.0% | 0.0% | 0.0% | 0.0% | 0.0% | 0.0% | 0.0% |
|  |  | 40% fT>MICx4 | 1g q8h | 9.0% | 11.4% | 11.6% | 11.7% | 11.7% | 11.7% | 11.9% |
|  | 9-hour Late  PIKRT^€^ daily | 40% fT>MIC | 500 mg q12h | 0.0% | 0.0% | 0.0% | 0.0% | 0.0% | 0.0% | 0.0% |
|  |  | 40% fT>MICx4 | 1g q8h | 0.0% | 0.3% | 0.5% | 0.5% | 0.5% | 1.0% | 0.7% |
| 5 | Extended PIKRT  daily | 40% fT>MIC | 500 mg q8h | 0.0% | 0.0% | 0.0% | 0.0% | 0.0% | 0.0% | 0.0% |
|  |  | 40% fT>MICx4 | 750 mg q6h | 0.5% | 1.0% | 1.0% | 1.0% | 1.0% | 1.0% | 1.0% |

KRT: kidney replacement therapy; PD: pharmacodynamic; MCS: Monte carlo simulation; HD: Hemodialysis at dialysate flow rate (Qd) 300 ml/min; UF: Ultrafiltration at ultrafilration flow rate of 5 ml/min; PIKRT: Prolonged intermittent kidney replacement therapy (9-hour PIKRT runs at Qd 100 ml/minl and extended PIKRT runs at Qd 50 ml/min for 24 hours); LD: loading dose

^†^60% fT>MIC or 60% fT>MICx4 denotes at least 60% of time during each day of one week of antibiotic therapy that the free plasma imipenem concentration was greater than the target minimum inhibitory concentration (MIC) or four time of the target MIC of 2 mg/L (susceptibility breakpoint MIC for *P. aeruginosa*).

^£^This indicates the percentage of 5000 simulated patients that were at or above the suggested ceftazidime safety threshold (i.e. free plasma concentration ≥ MICx8 = 16 mg/L) at the end of each day during 1 week of therapy.

^€^ EARLY PIKRT is where the initial imipenem dose is infused at the beginning of 9-hour PIKRT and late PIKRT where the initial imipenem dose is given 15 hours prior to 9-hour PIKRT virtual patients receiving each of 5 kidney replacement therapy.

**Table S39. Probability of Neurotoxicity of MCS-driven Meropenem Dosing Recommendation in Five Kidney Replacement Therapies.**

|  | KRT setting | PD target^†^ | MCS-driven Meropenem dosing recommendation | Probability of total concentration above neurotoxicity threshold at the end of each day during 1 week of therapy^£^ | | | | | | |
| --- | --- | --- | --- | --- | --- | --- | --- | --- | --- | --- |
|  |  |  |  | Day 1 | Day 2 | Day 3 | Day 4 | Day 5 | Day 6 | Day 7 |
| 1 | 4-hour HD on Mon-Wed-Fri | 40% fT>MIC | 500 mg q24h post-HD | 0.0% | 0.0% | 0.0% | 0.0% | 0.0% | 0.0% | 0.0% |
|  |  | 40% fT>MICx4 | 1g LD, 500 mg q12h post-HD | 0.0% | 0.0% | 0.0% | 0.0% | 0.0% | 0.4% | 0.0% |
| 2 | 4-hour HD daily | 40% fT>MIC | 500 mg q24h post-HD | 0.0% | 0.0% | 0.0% | 0.0% | 0.0% | 0.0% | 0.0% |
|  |  | 40% fT>MICx4 | 1g LD, 500 mg q12h post-HD | 0.0% | 0.0% | 0.0% | 0.0% | 0.0% | 0.0% | 0.0% |
| 3 | Sequential 4-hour HD & 20-hour UF | 40% fT>MIC | 500 mg q24h post-HD | 0.0% | 0.0% | 0.0% | 0.0% | 0.0% | 0.0% | 0.0% |
|  |  | 40% fT>MICx4 | 1g LD, 500 mg q12h post-HD | 0.0% | 0.0% | 0.0% | 0.0% | 0.0% | 0.0% | 0.0% |
| 4 | Early 9-hour  PIKRT^€^  daily | 40% fT>MIC | 500 mg q12h | 0.0% | 0.0% | 0.0% | 0.0% | 0.0% | 0.0% | 0.0% |
|  |  | 40% fT>MICx4 | 500 mg q8h | 0.0% | 0.0% | 0.0% | 0.0% | 0.0% | 0.0% | 0.0% |
|  | Late 9-hour  PIKRT^€^ daily | 40% fT>MIC | 500 mg q12h | 0.0% | 0.0% | 0.0% | 0.0% | 0.0% | 0.0% | 0.0% |
|  |  | 40% fT>MICx4 | 500 mg q8h | 0.0% | 0.0% | 0.0% | 0.0% | 0.0% | 0.0% | 0.0% |
| 5 | Extended PIKRT | 40% fT>MIC | 500 mg q12h | 0.0% | 0.0% | 0.0% | 0.0% | 0.0% | 0.0% | 0.0% |
|  |  | 40% fT>MICx4 | 1g q12h | 0.0% | 0.0% | 0.0% | 0.0% | 0.0% | 0.0% | 0.0% |

KRT: kidney replacement therapy; PD: pharmacodynamic; MCS: Monte carlo simulation; HD: Hemodialysis at dialysate flow rate (Qd) 300 ml/min; UF: Ultrafiltration at ultrafilration flow rate of 5 ml/min; PIKRT: Prolonged intermittent kidney replacement therapy (9-hour PIKRT runs at Qd 100 ml/minl and extended PIKRT runs at Qd 50 ml/min for 24 hours); LD: loading dose

^†^40% fT>MIC or 40% fT>MICx4 denotes at least 60% of time during each day of one week of antibiotic therapy that the free plasma meropenem concentration was greater than the target minimum inhibitory concentration (MIC) or four time of the target MIC of 2 mg/L (susceptibility breakpoint MIC for *P. aeruginosa*).

^£^This indicates the percentage of 5000 simulated patients that were at or above the suggested meropenem safety threshold (i.e. total plasma concentration ≥ 64 mg/L) at the end of each day during 1 week of therapy.

^€^ EARLY PIKRT is where the initial meropenem dose is infused at the beginning of 9-hour PIKRT and late PIKRT where the initial meropenem dose is given 15 hours prior to 9-hour PIKRT virtual patients receiving each of 5 kidney replacement therapy.
